# The Role of Hematophagous Arthropods, Other than Mosquitoes and Ticks, in Arbovirus Transmission

**DOI:** 10.3390/v17070932

**Published:** 2025-06-30

**Authors:** Bradley J. Blitvich

**Affiliations:** Department of Veterinary Microbiology and Preventive Medicine, College of Veterinary Medicine, Iowa State University, 2116 Veterinary Medicine, Ames, IA 50011, USA; blitvich@iastate.edu; Tel.: +1-515-294-9861

**Keywords:** arbovirus, arthropod, hematophagous, transmission, vector competence

## Abstract

Arthropod-borne viruses (arboviruses) significantly impact human, domestic animal, and wildlife health. While most arboviruses are transmitted to vertebrate hosts by blood-feeding mosquitoes and ticks, a growing body of evidence highlights the importance of other hematophagous arthropods in arboviral transmission. These lesser-known vectors, while often overlooked, can play crucial roles in the maintenance, amplification, and spread of arboviruses. This review summarizes our understanding of hematophagous arthropods, other than mosquitoes and ticks, in arboviral transmission, as well as their associations with non-arboviral viruses. Thirteen arthropod groups are discussed: bat flies, blackflies, cimicids (bat bugs, bed bugs, and bird bugs), *Culicoides* midges, fleas, hippoboscid flies, lice, mites, muscid flies (including horn flies and stable flies), phlebotomine sandflies, tabanids (including deer flies and horse flies), triatomines, and tsetse flies. Some of these arthropods are regarded as known or likely arboviral vectors, while others have no known role in arbovirus transmission. Particular attention is given to species associated with arboviruses of medical and veterinary significance. As the burden of arboviruses continues to grow, it is critical not to overlook the potential contribution of these lesser-known vectors.

## 1. Arboviruses

The term arthropod-borne virus (arbovirus) is a non-taxonomic classification that encompasses a diverse group of viruses that are transmitted by bite to susceptible vertebrate hosts by hematophagous (blood-feeding) arthropods. According to the recent comprehensive arbovirus catalog compiled by Huang and colleagues, there are 460 species (615 species/subtypes) of arboviruses [1]. These viruses belong to multiple families, but the majority are classified within the *Flaviviridae*, *Nairoviridae*, *Peribunyaviridae*, *Phenuiviridae*, *Rhabdoviridae*, *Sedoreoviridae*, and *Togaviridae* [1,2,3]. A defining characteristic of arboviruses is their ability to replicate in both arthropod vectors and vertebrate hosts. Many arboviruses are zoonotic and have caused major disease outbreaks among humans, domestic animals, and wildlife [2,3,4,5,6,7,8].

The most prevalent arboviral diseases are transmitted by mosquitoes and ticks. Mosquito-borne arboviruses of global public health importance include all four serotypes of dengue virus (*Flaviviridae*), chikungunya virus (*Togaviridae*), Japanese encephalitis virus (*Flaviviridae*), West Nile virus (WNV; *Flaviviridae*), yellow fever virus (*Flaviviridae*), and Zika virus (*Flaviviridae*) [9,10,11,12,13,14]. Tick-borne arboviruses of regional concern include Crimean–Congo hemorrhagic fever virus (CCHFV; *Nairoviridae*) in Africa, Asia, and Europe; Kyasanur Forest disease virus (KFDV; *Flaviviridae*) in Asia; Powassan virus (*Flaviviridae*) in North America and Russia; Rift Valley fever virus (RVFV; *Phenuiviridae*) in Africa; severe fever with thrombocytopenia syndrome virus (SFTSV; *Peribunyaviridae*) in Asia; and tick-borne encephalitis virus (TBEV; *Flaviviridae*) in Europe and Asia [15,16,17,18,19]. All known arboviruses have RNA genomes, except for African swine fever virus (ASFV; *Asfarviridae*) [20]. Viruses that cannot replicate in arthropods but are mechanically transmitted by them (i.e., via contaminated mouthparts) are not considered to be arboviruses [21].

## 2. Hematophagous Arthropods and Vector Competence

Hematophagous arthropods are taxonomically diverse, belonging to multiple families and orders (Table 1). While some hematophagous arthropods transmit viruses to vertebrates, many have no confirmed role in virus transmission. Some hematophagous arthropods also transmit bacteria, protozoa, and helminths [22,23,24]. To be regarded as an epidemiologically important arboviral vector, an arthropod must meet several key criteria: (i) the virus must replicate to sufficient levels within the arthropod’s midgut, disseminate to the salivary glands, and be transmitted via saliva during blood-feeding; (ii) the arthropod and virus must co-occur temporally and spatially; (iii) the arthropod must feed on the relevant vertebrate host; and (iv) a significant proportion of the arthropod population must be found infected with the virus in natural settings [25,26,27].

**Table 1:** A list of all known hematophagous arthropods and their taxonomic classification.

Arthropod	Family	Order
Bat flies	*Nycteribiidae* and *Streblidae*	*Diptera*
Blackflies	*Simuliidae*	*Diptera*
^1^ Blowflies	*Calliphoridae*	*Diptera*
Cimicids	*Cimicidae*	*Hemiptera*
*Culicoides* midges	*Ceratopogonidae*	*Diptera*
Fleas	Multiple families	*Siphonaptera*
Hippoboscid flies	*Hippoboscidae*	*Diptera*
Lice	Multiple families	*Psocodea*
Mites	Multiple families	*Acariformes* and *Parasitiformes*
^1^ Mosquitoes	*Culicidae*	*Diptera*
Muscid flies	*Muscidae*	*Diptera*
Phlebotomine sandflies	*Psychodidae*	*Diptera*
Tabanids	*Tabanidae*	*Diptera*
^1^ Ticks	*Argasidae*, *Nuttalliellidae*, and *Ixodidae*	*Ixodida*
Triatomines	*Reduviidae*	*Hemiptera*
Tsetse flies	*Glossinidae*	*Diptera*

^1^ Not covered in this review.

Vector competence experiments are performed to measure the ability of an arthropod to support the replication and dissemination of an arbovirus and subsequent transmission of the virus to a susceptible host [27,28,29,30,31,32,33]. The arthropod is usually exposed to the virus by allowing it to feed upon a viremic animal or infectious artificial blood meal or by direct needle inoculation into the thorax. One limitation of the intrathoracic inoculation technique is that it bypasses the natural midgut barrier, a critical component of the natural infection route [34,35]. After an appropriate extrinsic incubation period, the arthropod is allowed to feed upon a naïve vertebrate host, which is then monitored for evidence of viral infection. The extrinsic incubation period is defined as the time it takes for a virus to disseminate from the midgut to the salivary glands of the arthropod [28,30]. Infection, dissemination, and transmission rates are typically defined as the percentage of engorged arthropods that contain virus in their midguts, secondary organs (i.e., legs or wings), and saliva, respectively. The transmission rate can also be defined as the percentage of infected arthropods that successfully transmit to virus to an uninfected vertebrate host.

Importantly, the detection of a virus in an arthropod is not evidence that the virus can replicate in the arthropod. An arthropod can acquire a virus through a blood meal (either in the field or laboratory) yet be refractory to viral replication. If a virus persists in an arthropod for no longer than one or a few days, viral replication most likely did not occur. An increase in viral titer or the amount of viral RNA in an arthropod over time is strong evidence of active viral replication [36].

The role of mosquitoes and ticks in arboviral transmission has been extensively reviewed elsewhere and will not be addressed here [5,37,38,39,40,41,42,43]. This review instead summarizes our current knowledge regarding the involvement of other hematophagous arthropods in arbovirus transmission because there is a growing body of evidence to suggest that some have an important role in arboviral transmission. Thirteen arthropod groups are covered: bat flies, blackflies, cimicids, *Culicoides* midges, fleas, hippoboscid flies, lice, mites, muscid flies, phlebotomine sandflies, tabanids, triatomines, and tsetse flies. With the exception of mosquitoes and ticks, the only hematophagous arthropods not included in this review are blowflies (*Calliphoridae*). While blowflies are primarily scavengers that feed upon decaying organic matter, some species feed on blood during their larval stage [44,45]. However, blowflies are excluded from this review because the majority are not blood-feeders, and none have been implicated in the biological transmission of arboviruses.

Each group of arthropods discussed in this review is evaluated based on its association with (i) recognized arboviruses, (ii) other vertebrate-infecting viruses, and (iii) viruses not known to infect vertebrates. For the purpose of this review, a “recognized arbovirus” refers to any virus listed as such in the recent arbovirus catalog compiled by Huang and colleagues, which integrates data from the Arbovirus Catalog, the arbovirus list in Section VIII-F of the *Biosafety in Microbiological and Biomedical Laboratories* (6th edition), Virus Metadata Resource of International Committee on Taxonomy of Viruses, and GenBank [1]. “Other vertebrate-infecting viruses” are non-arboviral viruses known to infect vertebrates or vertebrate cell cultures or for which there is evidence for viral replication due to the detection of virus-derived small RNAs in vertebrate cells [46]. “Non-vertebrate-infecting viruses” are those for which there is no evidence of vertebrate or vertebrate cell infection.

The arthropods discussed in this review can be separated into three categories based on their roles in arbovirus transmission:

1. Arthropods known or suspected to be epidemiologically important biological vectors of arboviruses, i.e., blackflies, cimicids, *Culicoides* midges, and phlebotomine sandflies. These arthropods have yielded multiple isolations of the relevant viruses in field studies and have transmitted the virus under experimental conditions, while arthropods from no other groups have been identified as major vectors.
2. Arthropods that may play a minor role in the biological transmission of arboviruses, i.e., fleas and mites. These arthropods have also yielded multiple isolations of relevant virus and have transmitted the virus under experimental conditions, but other arthropods have been identified as major vectors.
3. Arthropods where there is insufficient evidence to suggest that they are biological vectors of arboviruses, i.e., bat flies, hippoboscid flies, lice, muscid flies, tabanids, triatomines, and tsetse flies. None of these arthropods have been shown to transmit arboviruses under experimental conditions.

This review is important because it addresses a significant gap in the arbovirology literature by systematically evaluating the potential role of non-mosquito, non-tick hematophagous arthropods in arbovirus transmission. While these arthropods have received far less attention, growing evidence suggests that some may serve as important or overlooked vectors. This review provides a foundation for future research efforts aimed at understanding the broader ecological dynamics of arboviral transmission and improving disease surveillance and control strategies.

## 3. Bat Flies

Bat flies (*Nycteribiidae* and *Streblidae*) are wingless ectoparasites that feed exclusively on the blood of bats [47]. The *Nycteribiidae* contains at least 275 species across 21 genera, whereas *Streblidae* comprises approximately 227 species in 31 genera. Bat flies primarily inhabit tropical and subtropical regions, with nycteribiids occurring mainly in the Eastern Hemisphere and streblids in the Western Hemisphere. Bat flies are not considered biological vectors of viruses of medical or veterinary importance, although five viruses capable of replicating in vertebrate cells have been detected in these arthropods: dengue virus 2 (DENV2; *Flaviviridae*), Kaeng Khoi virus (KKV; *Perbunyaviridae*), Mahlapitsi virus (MAHLV; *Sedoreoviridae*), Nelson Bay reovirus (NBV; *Sedoreoviridae*), and Wolkberg virus (WBV; *Peribunyaviridae*) (Table 2) [48,49,50,51,52,53]. DENV2 and KKV are recognized arboviruses, but the others are not [1].

### 3.1. Recognized Arboviruses

DENV2 was detected by RT-PCR in bat flies (*Strebla wiedemanni* and *Trichobius parasiticus*) collected from common vampire bats (*Desmodus rotundus*) in Mexico [53]. DENV2 was also found in tissues from the infested vampire bats. KKV was originally isolated from a wrinkle-lipped free-tailed bat (*Chaerephon plicatus*; aka *Tadarida plicata*) in Thailand [54]. Subsequent isolations were made from bat flies (*Eucampsipoda sundaica*) parasitizing Leschenault’s rousette fruit bats (*Rousettus leschenaultii*) in China and from *C. plicatus* bats in Cambodia [48,51,55]. KKV has also been isolated from cimicids (discussed later in this review). There is insufficient evidence to support the role of bat flies as vectors of DENV2 or KKV because vector competence studies have not been conducted.

**Table 2:** Arboviruses and other vertebrate-infecting viruses identified in field-collected bat flies.

Virus	Species Name	Abbreviation	Family	Recognized Arbovirus	^1^ Experimental Evidence of Bat Fly-Borne Virus Transmission	Citation
Dengue virus 2	*Orthoflavivirus denguei*	DENV2	*Flaviviridae*	Yes	^2^ -	[53]
Kaeng Khoi virus	*Orthobunyavirus kaengkhoiense*	KKV	*Perbunyaviridae*	Yes	-	[48,51]
Mahlapitsi virus	*Orthoreovirus mahlapitsiense*	MAHLV	*Sedoreoviridae*	-	-	[49]
Nelson Bay reovirus	*Orthoreovirus nelsonense*	NBV	*Sedoreoviridae*	-	-	[52,56]
Wolkberg virus	*Orthobunyavirus wolkbergense*	WBV	*Peribunyaviridae*	-	-	[50]

^1^ Excludes mechanical transmission. ^2^ No.

### 3.2. Other Vertebrate-Infecting Viruses

MAHLV and WBV were originally isolated from bat flies (*Eucampsipoda africana*) collected from Egyptian fruit bats (*Rousettus aegyptiacus*) in South Africa, while NBV was originally isolated from a flying fox (*Pteropus poliocephalus*) in Australia [49,50,56]. Additional isolations of NBV were made from bat flies (*E. sundaica*) and bats (*R. leschenaultii*) in China [52]. All three viruses replicate in vertebrate cells and NBV causes disease in experimentally inoculated mice [49,50,52,56]. Additionally, a closely related NBV-like virus has been implicated in cases of acute respiratory distress in humans [57]. Vector competence experiments have not been performed to determine whether bat flies are capable of transmitting these viruses.

### 3.3. Viruses Not Known to Infect Vertebrates

Other viruses have been detected in bat flies using metagenomics, but none are known to replicate in vertebrate cells [58,59,60,61,62,63]. One example is Kanyawara virus (*Rhabdoviridae*), which was discovered in *Dipseliopoda* spp. bat flies parasitizing *Myonycteris* spp. bats in Uganda [59]. A subsequent study detected Kanyawara virus in an oral swab from an Angolan soft-furred fruit bat (*Lissonycteris angolensis*) infested with bat flies [58]. In the same study, the closely related Bughendera virus (*Rhabdoviridae*) was detected in a *Dipseliopoda* spp. bat fly. Additional novel viruses were detected in bat flies in China, Mexico, Nigeria, and Uganda [60,61,62,63]. In another study, novel rhabdoviruses were detected in bat flies (*Nycteribia kolenatii*, *Nycteribia schmidlii*, and *Penicillidia conspicua*) in Spain by RT-PCR using rhabdovirus-specific primers, but none have been demonstrated to replicate in vertebrate cells [64].

## 4. Blackflies

There are more than 2200 species of blackflies (*Simuliidae*) and most are hematophagous [65]. These arthropods occur on every continent, except Antarctica. Blackflies are vectors of several filarial nematodes and protozoan pathogens, including the etiological agent of human onchocerciasis (also known as river blindness) [66,67,68]. More pertinent to this review, blackflies harbor many viruses, including five recognized arboviruses: eastern equine encephalitis virus (EEEV; *Togaviridae*), Rift Valley fever virus (RVFV; *Phenuiviridae*), Venezuelan equine encephalitis virus (VEEV; *Togaviridae*), Vesicular stomatitis Indiana virus (VSIV; *Rhabdoviridae*), and Vesicular stomatitis New Jersey virus (VSNJV; *Rhabdoviridae*) (Table 3).

### 4.1. Recognized Arboviruses

Blackflies have been implicated as major biological vectors of vesicular stomatitis (VS), an agriculturally important disease of hoofed livestock in the Americas [69,70,71,72]. VS is caused by VSNJV and VSIV, known collectively as vesicular stomatitis virus (VSV). VSV has been frequently detected in blackflies during epizootics, with several studies reporting high prevalence rates [73,74,75,76,77,78,79]. For example, VSV was detected in 16 of 319 pools containing 1215 blackflies in California, the U.S. [75]. Blackflies of at least four species were positive for the virus: *Simulium argus*, *Simulium hippovorum*, *Simulium tescorum*, and *Simulium vittatum* complex. Infection rates were highest for *S. hippovorum* and *S. vittatum* complex. Additionally, VSV was detected in 11 of 77 pools of blackflies in New Mexico, the U.S. [77]. Over half of the positive pools contained *Simulium mediovittatum*, while the others contained *Simulium meridionale*, *Simulium notatum/griseum*, *Simulium robynae*, and an unidentified *Simulium* spp. In contrast, there was a low prevalence of VSV in blackflies in Chiapas, Mexico [80]. In this study, hematophagous arthropods (blackflies, midges, mosquitoes, and sandflies) were collected on cattle ranches with a history of VS cases. VSV was detected in 102 of 874 pools, but most positive pools contained mosquitoes and sandflies. Only one VSV-positive blackfly pool was detected, indicating that these arthropods are not major vectors of VSV in the study area.

Some species of blackflies are competent vectors of VSV [81,82,83,84,85,86]. In one study, 70% of colonized *Simulium vittatum* intrathoracically inoculated with VSNJV contained virus in their saliva at 10 days post-inoculation [85]. Following oral exposure, 45% of *S. vittatum* had detectable VSNJV in their saliva. Field-collected *Simulium bivittatum, Simulium longithallum*, and *S. vittatum* from Arizona, the U.S. were orally challenged with VSNJV strains from both Arizona and Oaxaca, Mexico [84]. VSNJV was detected in the saliva of 23% and 26% of *S. notatum* exposed to the Oaxaca and Arizona strains, respectively; however, neither viral strain disseminated to the salivary glands of *S. bivittatum* or *S. longithallum*. VSIV was also detected in the saliva of both colonized and wild *S. notatum* and *S. vittatum* at 10 days post-oral challenge [86]. Colonized *S. vittatum* intrathoracically inoculated with VSNJV successfully transmitted the virus to mice, pigs, and cattle [81,82,83]. Additionally, VSNJV was transmitted to uninfected *S. vittatum* co-feeding on the same nonviremic mice as infected blackflies [87].

EEEV, RVFV, and VEEV have occasionally been detected in blackflies. EEEV was isolated from one pool each of *Simulium johannseni* (aka *Eusimulium johannseni*) and *Simulium meridionale* in Wisconsin, the U.S. [88]. RVFV was isolated from at least one pool of *Simulium* blackflies (species not specified) in South Africa [89]. In Colombia, VEEV was isolated from 11 pools of blackflies (*Simulium cauicumn*, *Simulium exiguuma*, *Simulium metallicum*, *Simulium mexicanuma*, and *Simulium payneih*) [90]. Vector competence experiments have not been performed to determine whether blackflies are capable of transmitting any of these viruses.

**Table 3:** Arboviruses and other vertebrate-infecting viruses identified in field-collected blackflies.

Virus	Species Name	Abbreviation	Family	Recognized Arbovirus	^1^ Experimental Evidence of Blackfly-Borne Virus Transmission	Citation
Eastern equine encephalitis virus	*Alphavirus eastern*	EEEV	*Togaviridae*	Yes	^2^ -	[88]
Hepatitis B virus	*Orthohepadnavirus hominoidei*	HBV	*Hepadnaviridae*	-	-	[91]
Rift Valley fever virus	*Phlebovirus riftense*	RVFV	*Phenuiviridae*	Yes	-	[89]
Venezuelan equine encephalitis virus	*Alphavirus venezuelan*	VEEV	*Togaviridae*	Yes	-	[90]
Vesicular stomatitis Indiana virus	*Vesiculovirus indiana*	VSIV	*Rhabdoviridae*	Yes	-	[78]
Vesicular stomatitis New Jersey virus	*Vesiculovirus newjersey*	VSNJV	*Rhabdoviridae*	Yes	Yes (transmission to mice, pigs, and cattle)	[73,75,80,81,82,83]

^1^ Excludes mechanical transmission. ^2^ No.

### 4.2. Other Vertebrate-Infecting Viruses

Blackflies (*Simulium buissoni*) collected on the Marquesas Islands were tested by PCR for hepatitis B virus (HBV; *Hepadnaviridae*) [91]. HBV DNA was detected on the external surfaces of blackflies, but not within their internal tissues, indicating that these arthropods are refractory to HBV infection.

### 4.3. Viruses Not Known to Infect Vertebrates

Multiple DNA viruses were discovered in blackflies (*Austrosimulium australense*) from New Zealand using viral metagenomics [92]. The majority of the viruses were assigned to the family *Microviridae*, although viruses from the *Circoviridae* and *Genomoviridae* were also detected. None of the identified viruses are known to replicate in vertebrate cells. Two other DNA viruses identified in blackflies are invertebrate iridescent viruses 22 and 25 (*Iridoviridae*) [93,94]. Both viruses were originally isolated from blackflies in Wales and are assumed to have invertebrate-specific hosts. Because nearly all recognized arboviruses have RNA genomes, it is unlikely that the aforementioned viruses are unrecognized arboviruses.

## 5. Cimicids

The genus *Cimex* (family *Cimicidae*) contains 23 species of wingless ectoparasites that occur worldwide and feed exclusively on the blood of endotherms [95,96,97]. Two cimicid species (*Cimex lectularius* and *Cimex hemipterus*) exhibit a preference for human hosts and are commonly referred to as bed bugs. Others prefer bats or birds and are known as bat bugs and bird bugs, respectively. There is no evidence to suggest that cimicids transmit arboviruses to humans, although they are known to harbor many vertebrate-infecting viruses, including five recognized arboviruses: Fort Morgan virus (FMV; *Togaviridae*), Buggy Creek virus (BCRV; *Togaviridae*), KKV, Tonate virus (TONV; *Togaviridae*), and Usutu virus (USUV; *Flaviviridae*) (Table 4).

### 5.1. Recognized Arboviruses

Bird bugs, specifically cliff swallow bugs (*Oeciacus vicarius*), are suspected—but not conclusively confirmed—to be epidemiologically important vectors of FMV [98]. These ectoparasites primarily feed on nesting cliff swallows (*Petrochelidon pyrrhonota*), but also opportunistically feed on house sparrows (*Passer domesticus*), which frequently often occupy the nests of cliff swallows [99]. FMV was originally isolated from cliff swallow bugs, cliff swallows, and house sparrows in Colorado, the U.S. [100,101]. In a subsequent study, FMV was isolated from 80 of 1156 (6.9%) nestlings [102]. The prevalence of FMV in cliff swallow bugs is not well documented, although 75 isolates (38 alphaviruses and 37 apparent alphaviruses) were recovered from 5268 cliff swallow bugs (452 pools) collected in Colorado, with an unspecified number later identified as FMV [100,101].

Cliff swallow bugs are susceptible to FMV replication and dissemination and can efficiently transmit the virus to uninfected birds under laboratory conditions [103]. Eighteen days after feeding on a viremic bird, 80% of bugs were infected with FMV. At 24 days post-feeding, salivary glands were removed from 30 bugs and pooled (five pairs of glands in each pool). FMV was detected in five of six pools, and the transmission rate was estimated as 30%. At 47 days post-feeding, 16 bugs (in eight groups of two) were allowed to feed upon uninfected house sparrows (one bird per pair). Four birds became viremic and the transmission rate was estimated as 29%. Additionally, bugs were capable of transmitting FMV at 25, 33, 50, and 83 days post-infectious blood meal. There is no evidence to suggest that any other arthropods are competent vectors of FMV. Despite strong evidence indicating that cliff swallow bugs are likely to be the principal vectors of FMV, they cannot be classified as epidemiologically important vectors without field data demonstrating consistent FMV infection in wild populations.

BCRV (a subtype of FMV) was originally isolated from cliff swallow bugs in Oklahoma, the U.S. [104,105]. Subsequent isolations have been made from cliff swallow bugs, cliff swallows, and house sparrows at various locations across the U.S. [106,107,108,109,110,111]. In one study, BCRV was detected in 1459 of 7421 (19.7%) pools of cliff swallow bugs collected from active cliff swallow nests in Nebraska, the U.S. [106] BCRV causes clinical signs and, in some cases, fatal encephalitis in naturally infected house sparrows, whereas cliff swallows exhibit no apparent adverse effects [112]. Although cliff swallow bugs are known to harbor BCRV, vector competence experiments have not been conducted to determine whether they can transmit the virus to susceptible birds.

Other cimicid-associated arboviruses are KKV, TONV, and USUV (Table 4). KKV was isolated from bat bugs (*Stricticimex parvus* and *Cimex insuetus*) in Thailand [113]. Additional isolations have been made from bat flies and bats (discussed earlier in this review) [48,51,54,55,113]. However, KKV failed to replicate in experimentally inoculated bat bugs [114]. TONV has been isolated from cliff swallow bugs, mosquitoes, cliff swallows, house sparrows, and a fringe-lipped bat (*Trachops cirrhosus*) in the U.S. and South America [115,116,117,118]. TONV is clinically significant because it has been associated with fatal encephalitis and fetal abnormalities in humans [119,120,121]. However, cliff swallow bugs appear to be inefficient biological vectors of TONV. Only 2 of 124 (1.6%) bugs became infected after feeding upon a viremic bird, while 5 of 11 (45.5%) *Culex tarsalis* mosquitoes became infected [116]. USUV was detected in one of 96 pools of house martin bugs (*Oeciacus hirundinis*) collected from abandoned house martin (*Delichon urbicum*) nests in the Czech Republic [122]. Vector competence experiments have not been performed to determine whether bird bugs are capable of biologically transmitting USUV.

### 5.2. Other Vertebrate-Infecting Viruses

Field-collected *C. hemipterus* and *C. lectularius* have tested positive for HBV and hepatitis C virus (HCV; *Flaviviridae*), but laboratory studies suggest that bed bugs cannot biologically transmit these viruses or human immunodeficiency virus (*Retroviridae*) [123,124,125,126,127,128,129,130,131,132]. Avian paramyxovirus type 4 (*Paramyxoviridae*) and hepatitis E virus (*Hepeviridae*) have also been detected in field-collected bed bugs, while poliovirus (*Picornaviridae*) persisted for 7 days in *C. lectularius* that had fed upon viremic monkeys under laboratory conditions, although none of these viruses are known to be transmitted by bed bugs [133,134,135].

**Table 4:** Arboviruses and other vertebrate-infecting viruses identified in field-collected cimicids.

Virus	Species Name	Abbreviation	Family	Recognized Arbovirus	^1^ Experimental Evidence of Efficient Cimicid-Borne Virus Transmission	Citation
Avian paramyxovirus type 4	*Paraavulavirus hongkongense*	APMV-4	*Paramyxoviridae*	^2^ -	-	[134]
Buggy Creek virus (a subtype of Fort Morgan virus)	*Alphavirus fortmorgan*	BCRV	*Togaviridae*	Yes	-	[104,105,106]
Fort Morgan virus	*Alphavirus fortmorgan*	FMV	*Togaviridae*	Yes	Yes (transmission to birds)	[100,101,103]
Hepatitis B virus	*Orthohepadnavirus hominoidei*	HBV	*Hepadnaviridae*	-	-	[127,129,132]
Hepatitis C virus	*Hepacivirus hominis*	HCV	*Flaviviridae*	-	-	[128]
Hepatitis E virus	*Paslahepevirus balayani*	HEV	*Hepeviridae*	-	-	[133]
Kaeng Khoi virus	*Orthobunyavirus kaengkhoiense*	KKV	*Perbunyaviridae*	Yes	-	[113]
Tonate virus	*Alphavirus tonate*	TONV	*Togaviridae*	Yes	-	[116]
Usutu virus	*Orthoflavivirus usutuense*	USUV	*Flaviviridae*	Yes	-	[122]

^1^ Excludes mechanical transmission. ^2^ No.

### 5.3. Viruses Not Known to Infect Vertebrates

Metagenomic surveys of European bat bugs (*Cimex pipistrelli*) have identified several viruses, but all are phylogenetically distinct from known vertebrate-infecting viruses [136]. Similarly, metagenomics studies of bed bugs have revealed numerous viruses, but with the exception of HCV, all are phylogenetically distinct from viruses known to replicate in vertebrates [128,136,137,138,139]. A *Sedoreoviridae*-like virus was also detected in field-collected bed bugs using electron microscopy, but there is no evidence indicating its ability to replicate in vertebrate cells [140].

## 6. Culicoides Midges

Hematophagous midges (*Ceratopogonidae*) have a worldwide geographic distribution and are well-known nuisance biters, often causing significant discomfort in humans and other vertebrates. The family contains more than 5400 species across 125 genera [141,142]. Many species in the genus *Culicoides* are vectors of viruses, protozoans, and bacteria [143,144,145]. Some *Culicoides*-associated viruses are arboviruses of medical and veterinary significance, and notable examples include African horse sickness virus (AHSV; *Sedoreoviridae*), Akabane virus (AKAV; *Peribunyaviridae*), bluetongue virus (BTV; *Sedoreoviridae*), bovine ephemeral fever virus (BEFV; *Rhabdoviridae*), epizootic hemorrhagic disease virus (EHDV; *Sedoreoviridae*), Main Drain virus (MDV; *Peribunyaviridae*), Oropouche virus (OROV; *Peribunyaviridae*), and Schmallenberg virus (SBV; *Peribunyaviridae*) [146,147,148,149,150,151,152,153,154,155,156,157] (Table 5).

### 6.1. Recognized Arboviruses

AHSV is the etiological agent of African horse sickness, a highly infectious and often fatal disease of equids endemic to sub-Saharan Africa, but also present in parts of Europe and Asia [146,148,151]. AHSV exists as nine antigenic types, designated as AHSV-1 to AHSV-9. *Culicoides imicola* is likely the principal vector of AHSV due to its abundance in endemic regions, its history of yielding more AHSV isolations than any other arthropod species, and its susceptibility to infection under laboratory conditions [54,158,159,160,161,162]. However, other *Culicoides* species, including *Culicoides bolitinos*, have also been implicated as vectors [161,163]. Field studies have reported the detection of AHSV in *Culicoides* midges [54,158,159,160,161]. For example, AHSV was detected in 12 of 95 pools of *Culicoides* midges from South Africa [160]. Of the AHSV-positive pools, seven pools consisted exclusively of *C. imicola*, four pools contained a mixture of *C. imicola* and other species, and one pool contained a mixture of species, excluding *C. imicola*. In a study performed in Spain, AHSV was isolated from six of 31 pools of *Culicoides* midges, including four pools of *C. imicola* [158]. A six-year surveillance study performed in South Africa reported the isolation of AHSV from 66 of 4506 pools of *Culicoides* midges [159]. All isolations were made from *C. imicola*.

Several studies have investigated the vector competence of *Culicoides* midges for AHSV [162,164,165]. Wetzel and colleagues were unable to demonstrate transmission of AHSV by *Culicoides* midges to horses [165]. The experiments were performed using field-collected *Culicoides* midges of multiple species, but most were *C. imicola* (aka *C. pallidipennis*). The same article refers to an unpublished study, cited via a personal communication, in which field-collected *Culicoides* midges (species not specified) were allowed to feed upon a horse infected with AHSV. Twelve days later, the midges were permitted to feed upon an uninfected horse, which became ill and died of African horse sickness 12 days later. In another study, *Culicoides variipennis* that had fed upon AHSV-infected, embryonated chicken eggs were able to transmit the virus to uninfected eggs [164]. In a more recent study, 17 species of *Culicoides* midges from South Africa were orally challenged with AHSV-3, AHSV-5, and AHSV-8, then tested for virus after a 10-day extrinsic incubation period [162]. AHSV-5 was detected in 8.5% of *C. imicola* and 20.6% of *C. bolitinos* but was not detected in any other species. AHSV-8 was detected in 26.8% of *C. imicola* and 1.7% of *C. bolitinos*, while all other species tested negative. ASHV-3 was found in 3.8% of *C. bolitinos*, but was not detected in any other species, including *C. imicola*.

AKAV is a teratogenic pathogen affecting cattle, sheep, and goats across Asia, Africa, Australia, and the Middle East [145,147]. In Australia, *Culicoides brevitarsis* is suspected to be the primary arthropod vector [166,167,168,169,170,171,172]. AKAV was isolated from 3 of 101 pools containing 5314 *C. brevitarsis* temporally and spatially associated with sentinel cattle, from which isolations were made in Queensland [166]. Laboratory studies have shown that AKAV disseminates to the salivary glands of *C. brevitarsis*, but transmission experiments were not performed [172]. In Japan, *Culicoides oxystoma* is implicated as the major arthropod vector [173,174,175,176]. In one study, AKAV was isolated from 3 of 84 pools of *Culicoides* spp. collected inside a cowshed where calves had seroconverted [174]. All positive pools contained *C. oxystoma.* AKAV has also been isolated from *Culicoides* midges in China, although the species were not identified [177]. The vector competence of *C. oxystoma* and five other *Culicoides* spp. from Japan has been evaluated [176]. All species were susceptible to oral infection, but viral titers were significantly higher in *C. oxystoma*. In Africa and the Middle East, *C. imicola* is suspected to be the primary vector of AKAV, although vector competence experiments have not been performed [178,179,180,181]. Vector competence has been assessed for *Culicoides nubeculosus* and *Culicoides variipennis*, which do not occur in the endemic range of AKAV [182]. Following intrathoracic inoculation, the virus persisted for at least nine days in both species, but only *C. variipennis* was susceptible to oral infection and successfully transmitted the virus through a membrane at 7–10 days post-infection.

BTV is the etiological agent of bluetongue disease, a non-contagious disease affecting domestic and wild ruminants in Africa, the Middle East, Asia, Australia, the Americas, and Europe [149,183,184]. There are at least 36 recognized serotypes of BTV, designated as BTV-1 to -36 [185]. Most serotypes are primarily transmitted by *Culicoides* spp. midges, but some are transmitted horizontally by direct contact between vertebrate hosts. In North America, *Culicoides sonorensis* is considered the primary arthropod vector of BTV, although other species, i.e., *Culicoides insignis* and *Culicoides stellifer*, may also contribute to transmission [152]. In the Caribbean and South America, *C. insignis* is recognized as an important vector [186,187]. In Australia, *C. brevitarsis* is regarded as the principal vector, although several less-widespread species may have regionally important roles [188]. In Africa, the primary vector is *C. imicola*, although *C. bolitinos* and select other species may also be involved in transmission [189]. *C. imicola* is also a major vector in southern Europe and the Mediterranean region, while *Culicoides obsoletus* and *Culicoides pulicaris* are important in northern Europe [190]. In Asia, the major vectors of BTV are not well defined, but several *Culicoides* species, including *Culicoides actoni*, *Culicoides fulvus*, and *Culicoides wadai*, have been implicated [185,191,192].

Many field studies have reported the presence of BTV in *Culicoides* midges, and the prevalence has often been high [192,193,194,195,196,197,198,199,200]. In Italy, BTV RNA was detected in 68 of 77 pools comprising 1591 *Culicoides* spp. midges [196]. The prevalence of BTV was also high in *C. imicola*, with 52 of 53 pools comprising 1305 individuals testing positive. Similarly, in Morocco, BTV RNA was found in 45 of 55 pools containing 2003 *Culicoides* spp. midges [193]. *C. imicola* again showed a notably high prevalence with 15 of 16 pools testing positive. In Kazakhstan, BTV RNA was detected in 16 of 79 pools of midges [194].

Multiple studies have investigated the vector competence of *Culicoides* midges for BTV, revealing varying results depending on the midges spp. and BTV serotype that were used [152,196,201,202,203,204,205,206,207,208,209,210,211,212,213,214,215,216]. *C. sonorensis* from the United States could transmit a live-attenuated vaccine strain of BTV-1 from vaccinated to unvaccinated sheep [213]. Another study demonstrated that a single infected *C. sonorensis* could transmit BTV-1 to naïve sheep with an efficiency of 80–100% [214]. *C. nubeculosus* and *C. impunctatus* from England supported the replication of BTV-3 and BTV-4 after feeding upon viremic sheep [215]. BTV-1 was detected in the saliva of 53% of *C. brevitarsis* from Australia experimentally inoculated with the virus and viral transmission to a naïve sheep was documented [216]. In another study, 22.7 to 82.0% of *C. bolitinos* and 1.9 to 9.8% *C. imicola* from South Africa were susceptible to infection by BTV-1, -3, and -4 after oral challenge [217]. Dissemination rates as high as 57.6%, 56.0%, and 50.5% were reported for *C. imicola* from Italy after oral challenge with BTV-2, -4, and -8, respectively [202].

BEFV is the etiological agent of bovine ephemeral fever, an acute febrile illness that primarily affects cattle and water buffalo across Africa, Asia, Australia, and the Middle East [150,218]. Evidence suggests that BEFV is predominantly transmitted by mosquitoes and *Culicoides* midges, although evidence of *Culicoides*-borne viral transmission under laboratory conditions is lacking. BEFV has been isolated once from *C. brevitarsis* in Australia, once each from *C. imicola* and *Culicoides coarctatus* in Zimbabwe, and once from a pool containing several different species of *Culicoides* midges in Kenya [180,219,220]. BEFV has also been detected at least once in *C. puncticollis* in Turkmenistan, and BEFV RNA was detected by RT-PCR in one of 166 pools of *Culicoides* spp. midges in Korea [221,222]. However, BEFV has been isolated more often from mosquitoes. In one study, BEFV was isolated from 10 pools of *Aedes*, *Culex*, and *Uranotaenia* spp. mosquitoes in Australia [223]. Three more isolations were made from *Anopheles bancroftii* in Australia [219,224].

Several studies have investigated whether *Culicoides* midges are competent vectors of BEFV [225,226,227,228]. Colonized *C. sonorensis* and mosquitoes (*Aedes aegypti*, *Culex pipiens*, and *Culex quinquefasciatus*) were challenged with BEFV by artificial blood meal and intrathoracic inoculation [225]. After oral exposure, infection and dissemination rates in *C. sonorensis* were 7.6% and 1.2%, respectively, while no evidence of viral replication was observed in any mosquitoes. BEFV replication occurred in all *C. sonorensis* challenged by intrathoracic inoculation, although the midges failed to transmit the virus to naïve calves. In an earlier study, BEFV was isolated from one of 526 (0.2%) *C. brevitarsis* and an unspecified number of *Culicoides marksi* 10 days after oral challenge [226]. BEFV was also isolated from 3 of 23 (13.0%) *Culex annulirostris* that had been orally challenged with the virus 12 days earlier.

EHDV is the etiological agent of epizootic hemorrhagic disease, a significant cause of morbidity and mortality among livestock and wildlife across North and South America, Africa, Asia, Australia, and the Middle East [157,185,229]. The virus occurs as seven serotypes, designated as EHDV-1 to -7. The *Culicoides* spp. implicated in the transmission of EHDV include *C. imicola*, *C. sonorensis*, *C. obsoletus*, *C. brevitarsis*, *C. insignis*, *C. mohave*, *C. oxystoma,* and *C. variipennis*. All of these species have tested positive for EHDV in surveillance studies [219,230,231,232,233,234]. For example, EHDV was detected by RT-PCR in 6 of 103 pools containing 964 *Culicoides* midges collected during an EHDV outbreak at the Minnesota Zoo in the U.S., with half the positive pools comprising *C. sonorensis* [230]. In Italy, 18 of 411 pools containing 5721 *Culicoides* midges contained EHDV RNA, including 10 pools of *C. imicola* [232].

Vector competence experiments have shed light on the role of *Culicoides* midges in EHDV transmission [152,235,236,237,238]. In one study, *C. sonorensis* midges were orally exposed two strains of EHDV-2, resulting in infection, dissemination, and transmission rates as high as 100%, 90%, and 79%, respectively, for both strains at 9 to 14 days after exposure [235]. Transmission rates were calculated by dividing total midges with detectable virus in saliva by total midges with disseminated infection. Additional studies have investigated the ability of *Culicoides* midges to experimentally transmit EHDV to vertebrate animals [236,238,239]. In one such experiment, *C. sonorensis* were allowed to feed on EHDV-2 inoculated deer and then, after an extrinsic incubation period of 14 to 16 days, permitted to feed on a naïve deer, which subsequently developed viremia and severe disease [240]. Biological transmission of EHDV-1 from one deer to another by *C. variipennis* also has been documented [239]. In another study, *C. sonorensis* became infected after feeding on embryonated chicken eggs inoculated with EHDV-1, -2, and -6; however, none successfully transmitted the virus to uninfected eggs [238].

MDV occurs in the western U.S. and is not a common cause of disease, although it has been isolated from the brain of a horse that died of encephalitis [156]. Most arthropod-derived isolations of MDV have been made from *Culicoides variipennis* (on at least 31 occasions) and undetermined *Culicoides* spp. (on at least 65 occasions) [241]. Experimental studies have shown that MDV replicates in both *C. variipennis* and *C. nubeculosus* after intrathoracic inoculation and oral ingestion [242]. Moreover, both species were capable of transmitting the virus to suspensions of uninfected blood suspensions 16 days after feeding upon infectious blood.

OROV occurs in Latin America and the Caribbean and is the etiological agent of Oropouche fever, the only known *Culicoides*-transmitted disease of humans [153,154,155,243,244]. *Culicoides paraeneses* is considered the primary urban vector because it is often highly abundant in areas where human outbreaks occur and the virus has been isolated from this species [245,246,247,248,249,250]. For instance, over 95% of *Culicoides* collected during an outbreak in Brazil in 1975 were identified as *C. paraeneses*, with two isolations of OROV made from the approximately 15,000 *Culicoides* inoculated into mice [246]. *C. paraensis* is a competent vector of OROV in the laboratory [251,252,253]. *C. paraensis* that had engorged upon viremic hamsters were able to transmit OROV to naïve hamsters after a 4 to 9 day extrinsic incubation period [252]. Viral transmission rates were calculated as 25% to 83%. In another study, OROV was experimentally transmitted from a viremic human to a hamster by *C. paraensis* after a 6 to 12 day extrinsic incubation period [251]. *C. sonorensis* is also a competent laboratory vector of OROV [254]. In this study, three species of arthropods (*C. sonorensis*, *Cx. quinquefasciatus*, and *Cx. tarsalis*) from the United States were provided OROV-spiked blood meals. At 14 days post-engorgement, *C. sonorensis* demonstrated infection, dissemination, and transmission rates of 87%, 83%, and 20%, while both mosquito species demonstrated transmission rates of <1%. Other studies have also shown that mosquitoes are incompetent vectors of OROV [255,256,257,258].

SBV is a cause of pregnancy loss and congenital malformations in domestic ruminants in Europe [259,260,261,262]. *Culicoides* midges, particularly those in the *Obsoletus* complex, appear to be major vectors. The *Obsoletus* complex contains as many as five species: *Culicoides chiopterus*, *Culicoides dewulfi*, *Culicoides montanus*, *Culicoides obsoletus*, and *Culicoides scoticus* [263,264,265]. SBV has been detected in multiple species of *Culicoides* midges [199,266,267,268,269,270,271,272,273,274,275,276,277]. In Belgium, SBV RNA was detected in 12 of 178 pools comprising 1976 *Culicoides* midges [267]. The positive pools consisted of *C. obsoletus* complex, *C. obsoletus*, *C. dewulfi*, *Culicoides chiopterus*, and *Culicoides pulicaris*. In France and Italy, SBV RNA was detected in 22 of 508 pools (5135 midges) and 3 of 759 pools (28,177 midges), respectively [266]. Most positive pools consisted of *C. obsoletus* complex and *C. obsoletus*. In the Netherlands, SBV RNA was detected in 12 of 230 pools of *C. obsoletus* complex [270].

Several studies have investigated whether *Culicoides* midges are susceptible to SBV infection [266,275,278,279]. SBV was detected in 8 of 147 (7.3%) *C. obsoletus* complex midges after an infectious artificial blood meal administered 8 days earlier [278]. In another study, colonized *C. nubeculosus* challenged via artificial blood meals were refractory to SBV infection [275]. In experiments performed with field-collected midges, SBV RNA was detected in the heads of 26 of 32 (81%) individual *C. imicola* following oral challenge 10 days earlier. However, experimental transmission of SBV to naïve animals via the bite of infected *Culicoides* midges has not been demonstrated.

Other arboviruses have been detected in *Culicoides* spp. midges (Table 5). Among them are many viruses belonging to the *Peribunyaviridae*, although most have been isolated from *Culicoides* on only a few occasions and none have been efficiently transmitted by midges under experimental conditions [54,145,175,224,280,281,282,283,284,285,286,287,288]. Other arboviruses have occasionally been identified in *Culicoides* and this includes viruses belonging to *Sedoreoviridae* and *Rhabdoviridae*, in addition to Barmah Forest (*Togaviridae*), EEEV, CCHFV, Israel turkey meningoencephalitis virus (*Flaviviridae*), RVFV, VEEV, VSNJV, and WNV [54,175,224,289,290,291,292,293,294,295,296,297,298,299,300,301,302,303,304,305,306,307,308,309]. The ability of *Culicoides* midges to support the replication and dissemination of some of these viruses has been assessed, revealing that they are predominantly refractory to infection [310,311,312].

### 6.2. Other Vertebrate-Infecting Viruses

Fowlpox virus (*Poxviridae*) was detected in *Culicoides arakawae* in South Korea [313]. *C. arakawae* transmitted the virus to naive chickens after a 7-day extrinsic incubation period [314]. However, the transmission of fowlpox virus by arthropods is assumed to be mechanical [315].

### 6.3. Viruses Not Known to Infect Vertebrates

Many novel viruses have been detected in *Culicoides* spp. midges using metagenomics [316,317,318,319,320,321,322,323,324,325,326]. In most of these studies, the abilities of the newly discovered viruses to replicate in vertebrate cell lines were not assessed. When virus isolation in vertebrate cell culture was attempted, no isolates were recovered.

**Table 5:** Arboviruses and other vertebrate-infecting viruses identified in field-collected *Culicoides* midges ^1^.

Virus	Species Name	Abbreviation	Family	Recognized Arbovirus	^2^ Experimental Evidence of *Culicoides*-Borne Virus Transmission	Citation
Adelaide River virus	*Ephemerovirus adelaide*	ARV	*Rhabdoviridae*	Yes	^3^ -	[54]
African horse sickness virus	*Orbivirus alphaequi*	AHSV	*Sedoreoviridae*	Yes	Yes (transmission to embryonated chicken and anecdotal evidence of transmission to a horse)	[54,158,159,160,161,164,165]
Aino virus	*Orthobunyavirus ainoense*	AINOV	*Peribunyaviridae*	Yes	-	[175]
Akabane virus	*Orthobunyavirus akabaneense*	AKAV	*Peribunyaviridae*	Yes	Yes (transmission through a membrane)	[175]
Banna virus	*Seadornavirus bannaense*	BAV	*Sedoreoviridae*	Yes	-	[302]
Barmah Forest virus	*Alphavirus barmah*	BFV	*Togaviridae*	Yes	-	[224]
Beatrice Hill virus	*Tibrovirus beatrice*	BHV	*Rhabdoviridae*	Yes	-	[224]
Belmont virus	*Orthobunyavirus belmontense*	BELV	*Peribunyaviridae*	Yes	-	[224]
Bivens arm virus (a subtype of Tibrogargan virus)	*Tibrovirus tibrogargan*	BAV	*Rhabdoviridae*	Yes	-	[300]
Bluetongue virus	*Orbivirus caerulinguae*	BTV	*Sedoreoviridae*	Yes	Yes (transmission to sheep)	[192,193,194,195,196,197,198,199,200,213,214]
Bovine ephemeral fever virus	*Ephemerovirus febris*	BEFV	*Rhabdoviridae*	Yes	-	[180,219,220]
Bunyip Creek virus	none	BCV	*Sedoreoviridae*	Yes	-	[303]
Buttonwillow virus	*Orthobunyavirus buttonwillowense*	BUTV	*Peribunyaviridae*	Yes	-	[285,296]
Chuzan virus	^4^ none	CHUV	*Sedoreoviridae*	-	-	[298]
Crimean-Congo hemorrhagic fever virus	*Orthonairovirus haemorrhagiae*	CCHFV	*Nairoviridae*	Yes	-	[290]
Curionopolis virus	*Curiovirus curionopolis*	CURV	*Rhabdoviridae*	Yes	-	[301]
Douglas virus (a subtype of Schmallenberg virus)	*Orthobunyavirus schmallenbergense*	DOUV	*Peribunyaviridae*	Yes	-	[280]
Eastern equine encephalitis virus	*Alphavirus eastern*	EEEV	*Togaviridae*	Yes	-	[291]
Epizootic hemorrhagic disease virus	*Orbivirus ruminantium*	EHDV	*Sedoreoviridae*	Yes	Yes (transmission to deer)	[231,239,327,328,329]
Eubenangee virus	*Orbivirus eubenangeense*	EUBV	*Sedoreoviridae*	Yes	-	[224]
Facey’s Paddock virus	*Faceyspaddock orthobunyavirus*	FPV	*Peribunyaviridae*	Yes	-	[224]
Fowlpox virus	*Avipoxvirus fowlpox*	FWPV	*Poxviridae*	-	-	[314]
Fukuoka virus	*Ledantevirus fukuoka*	FUKV	*Rhabdoviridae*	Yes	-	[308]
Ibaraki virus	none	IBAV	*Sedoreoviridae*	Yes	-	[175]
Ingwavuma virus	*Orthobunyavirus ingwavumaense*	INGV	*Peribunyaviridae*	Yes	-	[287]
Iquitos virus (a subtype of Oropouche virus)	*Orthobunyavirus oropoucheense*	IQTV	*Peribunyaviridae*	Yes	-	[145]
Israel turkey meningoencephalitis virus	*Orthoflavivirus israelense*	ITV	*Flaviviridae*	Yes	-	[54]
Itacaiunas virus	*Curiovirus itacaiunas*	ITAV	*Rhabdoviridae*	Yes	-	[301]
Jatobal virus	*Orthobunyavirus jatobalense*	JATV	*Peribunyaviridae*	Yes	-	[145]
Kimberley virus	*Ephemerovirus kimberley*	KIMV	*Rhabdoviridae*	Yes	-	[305]
Kotonkan virus	*Ephemerovirus kotonkan*	KOTV	*Rhabdoviridae*	Yes	-	[304]
Leanyer virus	*Orthobunyavirus leanyerense*		*Peribunyaviridae*	Yes	-	[224]
Lokern virus (a subtype of Main Drain virus)	*Orthobunyavirus kernense*	LOKV	*Peribunyaviridae*	Yes	-	[296]
Madre de Dios virus (a subtype of Oropouche virus)	*Orthobunyavirus oropoucheense*	MDDV	*Peribunyaviridae*	Yes	-	[145]
Main Drain virus	*Orthobunyavirus kernense*	MDV	*Peribunyaviridae*	Yes	Yes (transmission to uninfected blood suspensions)	[296]
Manzanilla virus	*Orthobunyavirus manzanillaense*	MANV	*Peribunyaviridae*	Yes	-	[145]
Ngaingan virus	*Hapavirus ngaingan*	NGAV	*Rhabdoviridae*	Yes	-	[309]
Oropouche virus	*Orthobunyavirus oropoucheense*	OROV	*Peribunyaviridae*	Yes	Yes (transmission to hamsters)	[246,251]
Palyam virus	*Orbivirus palyamense*	PALV	*Sedoreoviridae*	Yes	-	[299]
Peaton virus	*Orthobunyavirus peachesterense*	PEAV	*Peribunyaviridae*	Yes	-	[281,282]
Pintupo virus (a subtype of Oropouche virus)	*Orthobunyavirus oropoucheense*	PINTV	*Peribunyaviridae*	-	-	[251]
Rift Valley fever virus	*Phlebovirus riftense*	RVFV	*Phenuiviridae*	Yes	-	[289]
Sabo virus	*Orthobunyavirus saboense*	SABOV	*Peribunyaviridae*	Yes	-	[290]
Sango virus	*Orthobunyavirus sangoense*	SANV	*Peribunyaviridae*	Yes	-	[290]
Sathuperi virus (a subtype of Schmallenberg virus)	*Orthobunyavirus schmallenbergense*	SATV	*Peribunyaviridae*	Yes	-	[290]
Schmallenberg virus	*Orthobunyavirus schmallenbergense*	SBV	*Peribunyaviridae*	Yes	-	[199,266,267,268,269,270,271,272,273,274,275,276,277]
Shamonda virus (a subtype of Schmallenberg virus)	*Orthobunyavirus schmallenbergense*	SHAV	*Peribunyaviridae*	Yes	-	[290]
Shuni virus	*Orthobunyavirus shuniense*	SHUV	*Peribunyaviridae*	Yes	-	[290]
Simbu virus	*Orthobunyavirus simbuense*	SIMV	*Peribunyaviridae*	Yes	-	[145]
Sweetwater Branch virus	*Tibrovirus sweetwater*	SWBV	*Rhabdoviridae*	Yes	-	[307]
Thimiri virus	*Orthobunyavirus thimiriense*	THIV	*Peribunyaviridae*	Yes	-	[224]
Tibrogargan virus	*Tibrovirus tibrogargan*	TIBV	*Rhabdoviridae*	Yes	-	[306]
Tinaroo virus (a subtype of Akabane virus)	*Orthobunyavirus akabaneense*	TINV	*Peribunyaviridae*	Yes	-	[280]
Venezuelan equine encephalitis virus	*Alphavirus venezuelan*	VEEV	*Togaviridae*	Yes	-	[297]
Vesicular stomatitis New Jersey virus	*Vesiculovirus newjersey*	VSNJV	*Rhabdoviridae*	Yes	-	[296]
Wallal virus	*Orbivirus betamitchellense*	WALV	*Sedoreoviridae*	Yes	-	[224]
Warrego virus	*Orbivirus gammamitchellense*	WARV	*Sedoreoviridae*	Yes	-	[224]
Weldona virus (a subtype of Tete virus)	*Orthobunyavirus teteense*	WELV	*Peribunyaviridae*	Yes	-	[288]
West Nile virus	*Orthoflavivirus nilense*	WNV	*Flaviviridae*	Yes	-	[292]
Wongorr virus	*Orbivirus deltamitchellense*	WGRV	*Sedoreoviridae*	Yes	-	[224]

^1^ Not an exhaustive list. ^2^ Excludes mechanical transmission. ^3^ No. ^4^ Not recognized by the International Committee on Taxonomy of Viruses.

## 7. Fleas

Fleas (*Siphonaptera*) are small, wingless insects that feed on the blood of endothermic vertebrates, most commonly rodents. Over 2500 species of fleas across 19 families have been identified [330,331]. Fleas occur on every continent, except Antarctica, and are vectors of several bacterial pathogens of medical significance, most notably, *Yersinia pestis*, the causative agent of plague. These insects are not considered major vectors of arboviruses, although a minor role in the transmission of TBEV has been suggested (Table 6).

### 7.1. Recognized Arboviruses

TBEV has been isolated from several species of fleas associated with small wild mammals, including *Ctenocephalides* spp. fleas infesting European moles (*Talpa europaea*) in Slovakia [332,333]. Nonetheless, such isolations are rare compared to the numerous isolations made from ticks [334,335]. Laboratory studies have demonstrated that fleas are capable of transmitting TBEV under experimental conditions [336,337]. For example, mouse fleas (*Leptopsylla segnis*) that fed on viremic mice were able to transmit TBEV to uninfected mice at two and three days post-feeding [336]. TBEV persisted in some fleas for up to four days. In another study, TBEV persisted in northern rat fleas (*Nosopsyllus fasciatus*) for 24 h and oriental rat fleas (*Xenopsylla cheopis*) for six days [338]. A key limitation of these studies is that viral titers were not quantified, leaving uncertainty as to whether active viral replication occurred in the fleas.

### 7.2. Other Vertebrate-Infecting Viruses

Hantaan virus (HTNV; *Hantaviridae*) has been isolated from fleas (*Leptopsylla segnis* and *Monopsyllus anisus*) collected from the nests of HTNV-infected striped field mice (*Apodemus agrarius*) in China [339]. In experimental studies, HTNV persisted for no more than two days in *L. segnis* and *M. anisus* following ingestion of virus-spiked blood. These findings suggest that both species of fleas are refractory to HTNV infection. Another virus that has been detected in field-collected fleas is canine distemper virus (CDV; *Paramyxoviridae*), with the detections made by RT-PCR in fleas (*Ceratophyllus sciurorum*) collected from American mink (*Neovison vison*) during an outbreak of CDV on a mink farm in Denmark [340]. However, vector competence experiments have not been performed to determine whether fleas are capable of biologically transmitting CDV. Like CDV, feline leukemia virus (FeLV; *Retroviridae*) is a pathogen of veterinary significance [341,342]. Transmission experiments revealed that cat fleas (*Ct. felis*) that fed upon FeLV-infected blood could transmit the virus to uninfected blood [343]. However, the uninfected blood was offered immediately after the infectious blood meal, indicating that the transmission was mechanical. Notably, FeLV has never been isolated from naturally infected fleas. Fleas are also capable of transmitting feline calicivirus (FCV; *Caliciviridae*), a common pathogen of domestic cats [344]. FCV was isolated from cat fleas (*Ctenocephalides felis*) four days after they had engorged upon FCV-spiked blood. One of four kittens seroconverted after being fed upon by fleas that had engorged upon FCV-spiked blood. However, it is unclear whether biological transmission occurred because the length of time between blood meals was not specified. If the fleas fed upon the kittens shortly after their first blood meal, mechanical transmission most likely occurred. Isolations of FCV have never been made from naturally infected fleas.

**Table 6:** Arboviruses and vertebrate-infecting viruses identified in field-collected fleas.

Virus	Species Name	Abbreviation	Family	Recognized Arbovirus	^1^ Experimental Evidence of Flea-Borne Virus Transmission	Citation
Canine distemper virus	*Morbillivirus canis*	CDV	*Paramyxoviridae*	^2^ -	-	[340]
Hantaan virus	*Orthohantavirus hantanense*	HTNV	*Hantaviridae*	-	-	[339]
Tick-borne encephalitis virus	*Orthoflavivirus encephalitidis*	TBEV	*Flaviviridae*	Yes	Yes (transmission to mice, but it may have been mechanical)	[332,333,336,337]

^1^ Excludes mechanical transmission. ^2^ No.

The potential for fleas to support the replication of Marburg virus (MARV; *Filoviridae*) and Myxoma virus (*Poxviridae*) has been investigated, although neither virus has been detected in field-collected fleas [345,346]. MARV RNA was detected by quantitative RT-PCR in bat fleas (*Thaumapsylla breviceps*) that had fed upon viremic Egyptian fruit bats (*Rousettus aegyptiacus*), but the levels of viral RNA did not exceed the estimated amounts in the ingested blood, suggesting a lack of virus replication [345]. Furthermore, MARV-inoculated fleas failed to transmit the virus to uninfected bats. In a separate study, European rabbit fleas (*Sphilopsyllus cuniculi*) that had been immersed in a solution containing Myxoma virus successfully transmitted the virus to European rabbits (*Oryctolagus cuniculus*), but there is no evidence to indicate that transmission was biological [346].

### 7.3. Viruses Not Known to Infect Vertebrates

Eighteen novel viruses were detected by metagenomic analysis in fleas from three genera (*Stephanocircus*, *Pygiopsylla*, and *Macropsylla*) collected in Australia; however, none were closely related to viruses known to infect vertebrates [347]. In another metagenomic study, novel viruses from at least eight families were detected in fleas infesting bats (*Myotis daubentonii*, *Rhinolophus* spp., and *Rousettus leschenaultia*) in China, but none of the newly discovered viruses are known to infect vertebrates [63].

## 8. Hippoboscid Flies

Hippoboscid flies (*Hippoboscidae*), commonly referred to as louse flies and keds, are obligatory blood-feeders of birds and mammals [348,349]. At least 213 species of hippoboscid flies have been identified. These insects have a worldwide distribution but are most common in the tropics and subtropics. Hippoboscid flies have been implicated in the transmission of various bacterial and protozoan pathogens but are not regarded as significant vectors of viruses [348,350,351]. However, two recognized arboviruses and three other vertebrate-infecting viruses have been detected in these insects (Table 7).

### 8.1. Recognized Arboviruses

Two recognized arboviruses, BTV and WNV, have been detected in hippoboscid flies [352,353,354]. BTV was detected by RT-PCR in *Hippobosca equina* in Kazakhstan and WNV was detected by RT-PCR in *Icosta americana* in New Jersey, the U.S and Ontario, Canada. BTV has been experimentally transmitted to sheep by hippoboscid flies (*Melophagus ovinus*), but the mode of transmission was most likely mechanical [355]. There is no evidence to suggest that either BTV or WNV is biologically transmitted by hippoboscid flies.

### 8.2. Other Vertebrate-Infecting Viruses

Three additional vertebrate-infecting viruses have been detected in hippoboscid flies: Aksy-Durug Melophagus sigmavirus (*Rhabdoviridae*), border disease virus (*Flaviviridae*), and bovine viral diarrhea virus (*Flaviviridae*) [354,356,357]. Aksy-Durug Melophagus sigmavirus was detected in *M. ovinus* in Russia using viral metagenomics and shown to replicate in pig embryo kidney (PEK) cells, although it has not been isolated from any naturally infected vertebrates [357]. Border disease virus was identified in *M. ovinus* in China, and bovine viral diarrhea virus was identified in *H. equina* in Kazakhstan, but there is no evidence to suggest that hippoboscid flies are biological vectors of either virus [354,356].

**Table 7:** Arboviruses and other vertebrate-infecting viruses identified in field-collected hippoboscid flies.

Virus	Species Name	Abbreviation	Family	Recognized Arbovirus	^1^ Experimental Evidence of Hippoboscid-Borne Virus Transmission	Citation
Aksy-Durug Melophagus sigmavirus	^2^ none	ADMSV	*Rhabdoviridae*	^3^ -	-	[357]
Bluetongue virus	*Orbivirus caerulinguae*	BTV	*Sedoreoviridae*	Yes	-	[354]
Border disease virus	*Pestivirus ovis*	BDV	*Flaviviridae*	-	-	[356]
Bovine viral diarrhea virus	*Pestivirus bovis*	BVDV	*Flaviviridae*	-	-	[354]
West Nile virus	*Orthoflavivirus nilense*	WNV	*Flaviviridae*	Yes	-	[352,353]

^1^ Excludes mechanical transmission. ^2^ Not recognized by the International Committee on Taxonomy of Viruses. ^3^ No.

### 8.3. Viruses Not Known to Infect Vertebrates

Multiple viruses with apparent arthropod-specific host ranges have been detected in hippoboscid flies [357,358]. For example, a metagenomic analysis of *M. ovinus* in Russia resulted in the identification of four viruses from three families (*Iflaviridae*, *Reoviridae*, and *Solemoviridae*), none of which could replicate in vertebrate cells [357].

## 9. Lice

Lice are wingless ectoparasites belonging to multiple families within the order *Psocodea*, which comprises approximately 2500 species. Lice occur worldwide and are often classified into two major groups: sucking lice (superfamily Anoplura) and chewing lice (formerly the order *Mallophaga*, now but divided into three suborders: *Amblycera, Ischnocera, and Rhynchophthirina*) [359,360]. All sucking lice are obligate blood-feeders that parasitize mammals. Chewing lice (also known as biting lice) primarily parasitize birds, but also mammals, feeding on feathers, skin, fur, and scales. Although lice are not considered biological vectors of arboviruses, they transmit various bacterial pathogens of medical significance [361,362].

### 9.1. Recognized Arboviruses

No recognized arboviruses have been detected in naturally infected lice. However, KFDV was isolated from suckling lice (*Hoplopleura maniculata* and *Neohaematopinus echinatus*) that had fed on KFDV-inoculated jungle palm squirrels (*Funambulus tristriatus*) in the laboratory [363].

### 9.2. Other Vertebrate-Infecting Viruses

Southern elephant seal virus (SESV; *Togaviridae*) was originally isolated from hematophagous lice (*Lepidophthirus macrorhini*) infesting southern elephant seals (*Mirounga leonina*) in Australia [364]. Antibodies to SESV were detected in seals from which the lice were collected and the virus replicates in BHK-21 and Vero cells. Nonetheless, there is no evidence to suggest that lice are biological vectors of SESV. Similarly, there is also no evidence indicating that lice are biological vectors of poliovirus, as the virus was not recovered from sucking lice (*Pediculus capitis* and *Pediculus vestimenti*) that had fed upon viremic monkeys [135].

### 9.3. Viruses Not Known to Infect Vertebrates

Relatively little research has been conducted to identify and characterize the virome of lice [365,366,367,368,369]. Some studies have examined blood-feeding lice, while others investigated non-blood-feeding species. Most of the viruses identified to date are only distantly related to known vertebrate-infecting viruses

## 10. Mites

Mites are small arachnids belonging to two superorders (*Acariformes* and *Parasitiformes*) [370,371,372]. More than 48,200 species of mites have been identified, and they are globally distributed, occurring in almost every type of environment. Some mites feed upon the blood, skin, and keratin of vertebrates, while others feed on insects or plants. Mites are vectors of bacterial pathogens of medical significance, including the etiological agents of rickettsialpox and scrub typhus [373]. More pertinent to this review, mites harbor a diverse range of viruses (Table 8).

### 10.1. Recognized Arboviruses

Studies performed in the 1940s provide evidence that mites are biological vectors of St. Louis encephalitis virus (SLEV; *Flaviviridae*), but subsequent work could not verify these findings and *Culex* spp. mosquitoes are now considered the principal vectors of the virus. SLEV was isolated from naturally infected chicken mites (*Dermanyssus gallinae*) in Missouri, the U.S. and persisted for five months in naturally infected mites transferred to the laboratory for propagation [374,375]. *D. gallinae* became infected with SLEV after feeding upon viremic chickens and were able to transmit the virus to naïve chickens [376,377]. Naturally infected mites also transmitted the virus to naïve chickens. However, a later study reported that SLEV persists for only 2 h in *D. gallinae* [378]. *Ornithonyssus sylviarum* and *Ornithonyssus bursa* bird mites were also evaluated, with SLEV persisting for only 2 and 36 h, respectively. SLEV was not transmitted to naïve chickens by *D. gallinae*, *O. bursa*, and *O. sylviarum* that had fed upon viremic chickens one to three days earlier. Mites are now considered to have an inconsequential role in the maintenance of SLEV in nature because of the conflicting data produced in the transmission experiments, sparse number of SLEV-infected fleas collected in the field, and abundance of data revealing that *Culex* spp. mosquitoes are the principal vectors.

EEEV and Western equine encephalitis virus (WEEV; *Togaviridae*) have been isolated from mites in the U.S. [379,380,381,382,383]. Isolations of EEEV were made from chicken mites (*D. gallinae*) in Tennessee and isolations of WEEV were made from American bird mites (*Dermanyssus americanus*) in Colorado, chicken mites (*D. gallinae*) and tropical fowl mites (*Liponyssus bursa*) in Texas, and tropical rat mites (*Ornithonyssus sylviarum*; aka *Liponyssus sylviarum*) in California. However, very few isolations of EEEV and WEEV have been made from mites compared to mosquitoes [291,384,385].

Vector competence experiments have been performed to determine whether mites can transmit EEEV and WEEV [386,387]. In one study, mites (*D. gallinae*, *L. bursa*, and *L. sylviarum*) were allowed to feed upon chickens infected with EEEV or WEEV [386]. In most cases, virus persisted in the mites no longer than two days, but there were exceptions, i.e., EEEV was isolated from a pool of *D. gallinae* at 15 days post-engorgement. In large-scale transmission experiments where several thousand mites that had fed upon viremic chickens were allowed to feed upon 125 naive chickens, there were only two instances of transmission; one where EEEV was transmitted to a chicken by *D. gallinae* after a 26-day extrinsic incubation and another where WEEV was transmitted to a chicken by *D. gallinae* after a 13-day extrinsic incubation. The authors concluded that mites are not major vectors of EEEV or WEEV. In another study, *D. gallinae* fed upon chicks that had been inoculated with EEEV [387]. EEEV persisted in the mites for 30 days, but viral titers did not increase with time, indicating that virus replication had not occurred in the mites. Mites that had fed on viremic chickens were allowed to feed on naive chickens at 3, 7, 11, 15, or 30 days post-engorgement. A subset of mites that engorged 3 and 7 days earlier could transmit EEEV, but the other mites did not. Although transmission was documented, the apparent inability of EEEV to replicate in mites suggests that viral transmission was mechanical. Two other arboviruses, VEEV and Langat virus (*Flaviviridae*), are also incapable of replicating in mites under experimental conditions [388,389].

Cocal virus (*Rhabdoviridae*) was originally isolated from a pool of *Gigantolaelaps* mites infesting rice rats in Trinidad and has since been detected in other insects and livestock [390]. SFTSV was detected by RT-PCR in mites (*Laelaps echidninus*, *Leptotrombidium delicense*, and *Leptotrombidium scutellare*) in China [391,392]. In another study, SFTSV was detected by RT-PCR in mites (*Blankaartia acuscutellaris*, *Gahrliepia walchia*, *Leptotrombidium* spp., and *Schoengastia kanhaensis*) infesting rodents in Thailand [393]. Transmission experiments have not been performed to determine whether mites are competent vectors of Cocal virus or SFTSV.

### 10.2. Other Vertebrate-Infecting Viruses

HTNV is not a recognized arbovirus, but mites have been suggested to play a minor role in its biological transmission [394]. HTNV was isolated from four species of field-collected mites (*Eulaelaps stabularis*, *Haemolaelaps glasgowi*, *Laelaps cynognathus*, and *Leptotrombidium scutellare*) in China [339,395,396,397,398]. Transstadial and transovarial transmission of HTNV by mites of several species have been reported [396,397,399,400,401]. HTNV was shown to persist in *L. scutellare* for up to 115 days and *E. stabularis* and *H. glasgowi* for up to 168 days [397,400,401]. Experimental studies have demonstrated that mites are capable of transmitting HTNV to uninfected mice [395,397,400,402]. In one study, HTNV was isolated from tissues harvested from mice that had been fed upon by infected mites (*L. scutellare*) fifteen days earlier [402]. Junin virus (*Arenaviridae*) is another vertebrate-infecting virus that has been detected in mites, with isolations made from spiny rat mites (*Echinolaelaps echidninus*) in Argentina [403].

**Table 8:** Arboviruses and other vertebrate-infecting viruses identified in field-collected mites.

Virus	Species Name	Abbreviation	Family	Recognized Arbovirus	^1^ Experimental Evidence of Mite-Borne Virus Transmission	Citation
Cocal virus	*Vesiculovirus cocal*	COCV	*Rhabdoviridae*	Yes	^2^ -	[390]
Eastern equine encephalitis virus	*Alphavirus eastern*	EEEV	*Togaviridae*	Yes	Yes (transmission to chickens, but highly inefficient)	[383,387]
Hantaan virus	*Orthohantavirus hantanense*	HTNV	*Hantaviridae*	-	Yes (transmission to mice; a minor role in HTNV transmission has been suggested)	[339,395,396,397,398,400,402]
Junin virus	*Mammarenavirus juninense*	JUNV	*Arenaviridae*	-	-	[403]
Severe fever with thrombocytopenia syndrome virus	*Bandavirus dabieense*	SFTSV	*Phenuiviridae*	Yes	-	[393]
St. Louis encephalitis virus	*Orthoflavivirus louisense*	SLEV	*Flaviviridae*	Yes	Conflicting data on whether mites can transmit SLEV to chickens, although they are considered inconsequential vectors	[374,375,376,377,378]
Western equine encephalitis virus	*Alphavirus western*	WEEV	*Togaviridae*	Yes	Yes (transmission to chickens, but highly inefficient)	[379,380,381,382]

^1^ Excludes mechanical transmission. ^2^ No.

### 10.3. Viruses Not Known to Infect Vertebrates

Many novel viruses have been detected in mites using metagenomics, although none are known to replicate in vertebrates [63,404].

## 11. Muscid Flies

Muscid flies (*Muscidae*) are found on every inhabited continent and comprise approximately 9000 species and 190 genera [405]. Many muscid flies are not primarily hematophagous while others, i.e., the horn fly (*Hematobia irritans*) and stable fly (*Stomoxys calcitrans*), are obligate blood-feeders [405,406]. Hematophagous muscid flies are mechanical vectors of various bacterial pathogens but are not recognized arboviral vectors, although they are known to harbor various vertebrate-infecting viruses (Table 9).

### 11.1. Recognized Arboviruses

WNV was isolated from stable flies infesting viremic American white pelicans (*Pelecanus erythrorhynchos*) in Montana, the U.S. [407]. The authors speculated that stable flies provide a source of infection via ingestion or mechanical transmission. ASFV DNA was detected in stable flies (*Stomoxys calcitrans*) collected on domestic pig farms in Lithuania [408]. In another study, ASFV DNA persisted for three days in stable flies that had engorged upon infected blood [409]. Transmission of ASFV to pigs via the ingestion of infected stable flies has also been reported [410]. However, there is no evidence to support biological transmission of ASFV by stable flies. Isolations of VSIV were made from house flies (*Musca domestica*) in Colorado, the U.S., but these insects are generally non-hematophagous [74].

**Table 9:** Arboviruses and other vertebrate-infecting viruses identified in field-collected muscid flies ^1^.

Virus	Species Name	Abbreviation	Family	Recognized Arbovirus	^2^ Experimental Evidence of Muscid-Borne Virus Transmission	Citation
African swine fever virus	*Asfivirus haemorrhagiae*	ASFV	*Asfarviridae*	Yes	^3^ -	[408]
Bovine leukosis virus	*Deltaretrovirus bovleu*	BLV	*Retroviridae*	-	-	[411]
West Nile virus	*Orthoflavivirus nilense*	WNV	*Flaviviridae*	Yes	-	[407]

^1^ Excludes non-hematophagous species. ^2^ Excludes mechanical transmission. ^3^ No.

### 11.2. Other Vertebrate-Infecting Viruses

Bovine leukosis virus (*Retroviridae*) has been detected in field collected horn flies, but these arthropods were unable to transmit bovine leukosis virus from infected to uninfected cattle under natural grazing conditions [411]. In another study, stable flies were fed artificial blood meals containing porcine reproductive and respiratory syndrome virus (PRRSV; *Arteriviridae*) [412]. PSSRV was detectable by qRT-PCR in the flies for up to 3 days, but the amount of viral nucleic acid decreased with time, indicating a lack of viral replication. Infectious virus persisted in the flies for only 24 h, and PRRSV has not been isolated from field-collected stable flies.

### 11.3. Viruses Not Known to Infect Vertebrates

Drosophila melanogaster Nora virus (*Picornaviridae*) has been detected in colonized horn flies, but there is no evidence to suggest that the virus replicates in vertebrate cells [413]. Densoviruses have also been detected in horn flies [414].

## 12. Phlebotomine Sandflies

Phlebotomine sandflies are small, hematophagous, mostly nocturnal insects belonging to the subfamily *Phlebotominae* (*Psychodidae*), which contains 1060 species and subspecies [415]. These insects have a wide geographic distribution, with the majority distributed throughout the tropics and subtropics, although none have been reported on the Pacific Islands or New Zealand. Phlebotomine sandflies are of major medical and veterinary importance, primarily because they are the principal vectors of *Leishmania* protozoan parasites [416,417,418,419]. More relevant to this review, phlebotomine sandflies harbor numerous arboviruses, most of which belong to the *Phenuiviridae* and *Rhabdoviridae* (Table 10) [420,421].

### 12.1. Recognized Arboviruses

Multiple viruses from the genera *Phlebovirus* (*Phenuiviridae*) have been detected in phlebotomine sandflies, although most have been detected once or on a few occasions. Several sandfly-associated phleboviruses cause human disease, including Sandfly fever Naples virus (SFNV), Sandfly fever Sicilian virus (SFSV), and Toscana virus (TOSV) [422,423,424,425,426,427,428,429,430,431,432,433,434,435]. SFNV and SFSV are endemic in the Mediterranean region and parts of Asia, where they cause an incapacitating febrile illness, while TOSV is a relatively common cause of meningitis and encephalitis in the Mediterranean region.

SFNV has been isolated from many pools of *Phlebotomus* spp. sandflies [54,436,437,438,439]. For example, SFNV was recovered from 4 of 59 pools containing 33,100 *Phlebotomus* spp. sandflies in India [437]. One positive pool contained *P. papatasi*, while the remaining three were not identified to species. In another study, 11 isolates were recovered from 26,734 *Phlebotomus* spp. sandflies in India [436,437,438]. Most isolates were presumed to be from *P. papatasi*, which constituted 99.2% of the total collection. Similarly, SFSV has been isolated from multiple pools of *Phlebotomus* spp. sandflies [54,422,431,440,441,442]. For instance, SFSV was identified in 49 of 304 pools containing 12,485 *Phlebotomus* spp. sandflies in Iran [441]. There is limited information confirming the vector competence of *Phlebotomus* spp. sandflies for SFNV and SFSV. However, in a study performed during World War II, eight of nine U.S. soldiers experimentally inoculated with an unknown phlebovirus (likely SFNV or SFSV) developed febrile illness [443]. *P. papatasi* were allowed to feed upon the patients and, after an extrinsic incubation period of 8 to 18 days, were then permitted to feed upon two uninfected volunteers. One of these individuals developed a febrile illness 3–4 days later, providing evidence of sandfly-borne phlebovirus transmission.

TOSV has been isolated from multiple pools of *P. perniciosus* and *P. perfiliewi*, with additional isolations also made from other *Phlebotomus* spp. (*P. longicuspis*, *P. major*, *P. neglectus*, *P. papatasi*, *P. sergenti*, and *P. tobbi*) and select *Sergentomyia* spp. (*S. dentata* and *S. minuta*) [444,445,446,447,448,449,450,451]. In one study, TOSV was isolated from 37 of 232 pools containing 16,374 sand flies (*P. perniciosus* and *P. perfiliewi*) in Italy [447]. The ability of *P. perniciosus* to support the replication of TOSV has been evaluated [452]. TOSV was detected in 95% of *P. perniciosus* following intrathoracic inoculation 2 to 12 days earlier. Viral titers increased over time. After oral exposure with of TOSV, up to 51% of *P. perniciosus* contained the virus at 4 to 14 days post-challenge, with minimal change in viral titers over time. Dissemination and transmission rates were not determined. In the same study, the ability of *P. perniciosus* to support the replication of Adria virus (a subtype of Salehabad virus), which is another sandfly-associated phlebovirus, was also assessed, yielding similar results to those reported for TOSV.

In a more recent study, the vector competence of four species of sandflies (*Phlebotomus papatasi*, *Phlebotomus sergenti*, *Phlebotomus tobbi*, and *Sergentomyia schwetzi*) for TOSV was evaluated [453]. Sandflies were orally challenged with two viral strains (denoted as TOSV-A and -B, respectively). At eight days post-challenge, TOSV-A was not detected in any sandflies. Likewise, at eight days post-challenge, TOSV-B was not detected in any *P. papatasi* or *S. schwetzi*, while TOSV-B infection rates of 52.8% and 6.2% were reported in *P. tobbi* and *P. sergenti*, respectively. Dissemination rates among infected *P. tobbi* and *P. sergenti* were 94.7% and 100%, respectively. Studies have not been performed to determine whether sandflies can experimentally transmit TOSV.

The ability of sandflies to support the replication of two other phleboviruses, Ntepes virus and RVFV, has been investigated [454,455,456,457]. Ntepes virus occurs in Kenya and has been isolated from sandflies (*Phlebotomine* spp. and *Sergentomyia* spp.) on three occasions [458,459]. Infection rates in *P. duboscqi* orally challenged with Ntepes virus 6, 10, and 15 days earlier were 10.7%, 5.4%, and 3.4%, respectively. None of the sandflies contained virus in their legs or salivary glands [454]. RVFV is primarily transmitted by mosquitoes, although it has been detected on at least one occasion in sandflies in a pool of *Phlebotomus* spp. in Nigeria (Genbank Accession No. ON856673). The vector competence of five species of sandflies (*Lutzomyia longipalpis*, *Phlebotomus duboscqi*, *P. papatasi*, *P. sergenti*, and *S. schwetzi*) for RVFV has been investigated [455,456,457]. In one experiment, 72 of 145 (49.7%) *P. duboscqi* that fed upon a viremic hamster became infected, including eight (5.5%) that developed disseminated infections [457]. Six sandflies with disseminated infections were allowed to feed on naïve hamsters, and five (83.3%) transmitted the virus. In contrast, only 6 of 236 (2.5%) and none of 378 (0%) *L. longipalpis* challenged with RVFV by intrathoracic inoculation and oral ingestion, respectively, transmitted the virus to susceptible hamsters [456]. Dissemination rates of 6% to 14% were reported in *P. duboscqi*, *P. papatasi*, *P. sergenti*, and *S. schwetzi* that fed upon RVFV-infected hamsters [455]. Additionally, 14% of *P. papatasi* and 50% of *P. duboscqi*, but none of the *S. schwetzi*, challenged with RVFV by intrathoracic inoculation were able to transmit the virus to susceptible hamsters.

Aside from SFNV, SFSV, and TOSV, only four phleboviruses have been detected in ≥20 pools of phlebotomine sandflies: Aguacate virus, Arbia virus (a subtype of Salehabad virus), Punta Toro virus, and Saddaguia virus (a subtype of SFNV). Aguacate virus occurs in Panama, where it was isolated from 31 of 3455 pools containing 252,512 *Lutzomyia* spp. sandflies [460]. Thirty-five isolates of Arbia virus were obtained from an unknown number of pools containing 10,843 *P. perfiliewi* and *P. perniciosus* in Italy and Spain [54,451]. Punta Toro virus was isolated on 49 occasions in a study where 219,512 sandflies from Panama were tested for virus [54,461]. Most isolations were made from *Lutzomyia trapidoi*. Saddaguia virus was isolated from 23 of 135 pools containing 3992 sandflies of various species, although most of the sample population were *Phlebotomus perniciosus*, *Phlebotomus perfiliewi*, and *Phlebotomus longicuspis* [462]. Vector competence experiments have not been performed with any of these viruses.

Multiple viruses belonging to the genus *Vesiculovirus* (*Rhabdoviridae*) have been detected in phlebotomine sandflies (Table 10) [463,464]. However, only three of these viruses have been isolated from over five pools: Chandipura virus (CHPV), VSIV, and VSNJV. CHPV is a human pathogen that has caused large outbreaks of encephalitis in India but also occurs in parts of Africa [465,466]. As already noted, VSIV and VSNJV are livestock pathogens that occur in the Americas [70,465,466].

Several studies report the detection of CHPV in *Phlebotomus* spp. and *Sergentomyia* spp. sandflies [467,468,469,470,471,472]. For example, CHPV was detected in 2 of 29 pools containing 625 *Phlebotominae* spp. sandflies in India [467]. In another study, three of nine pools of 277 *Sergentomyia* spp. sandflies collected during an outbreak of CHPV in India were positive for the virus [469]. Vector competence experiments have investigated whether sandflies are capable of transmitting CHPV [473,474]. CHPV replication and dissemination occurred in *P. papatasi* after intrathoracic inoculation, and clinical signs were observed in one of four mice fed upon by the infected *P. papatasi* after a seven-day extrinsic incubation period [474]. In another study, 65% of *P. argentipes* became infected after oral exposure to CHPV [473]. *P. argentipes* challenged with CHPV by intrathoracic inoculation could transmit the virus to naive mice and the estimated minimum transmission rate was 32%. Notably, experiments assessing the ability of orally challenged sandflies to transmit CHPV have not been performed.

VSIV and VSNJV has been detected in multiple pools of *Lutzomyia* spp. sandflies [54,80,475,476]. Additionally, evidence suggests that sandflies are competent vectors of these viruses [475,477,478,479,480]. *Lutzomyia trapidoi* became infected with VSIV after feeding upon viremic hamsters and could transmit the virus to naive mice after a 3-to-5-day extrinsic incubation period [477]. *Lutzomyia shannoni* supported the replication of VSNJV after challenge by both intrathoracic inoculation and membrane feeding [479]. VSNJV-inoculated sandflies could transmit the virus to a naive hamster after a 3-day extrinsic incubation period. Orally challenged sandflies could transmit the virus to mice after a 6-day extrinsic incubation period. Transmission electron microscopy revealed the presence of VSNJV in the salivary glands of *L. shannoni* five to six days after oral challenge [480]. High titers of VSNJV were reported in naturally infected *L. shannoni* [475].

Vector competence experiments have been performed with several other sandfly-associated vesiculoviruses. Carajas and Maraba viruses replicated in *L. longipalpis* following intrathoracic inoculation [481]. However, replication of Carajas virus did not occur in *L. longipalpis* after oral challenge, while sandflies were not orally challenged with Maraba virus. Vesicular stomatitis Alagoas virus replication occurred in *L. longipalpis* following intrathoracic inoculation and the infected sandflies could transmit the virus to naïve mice [482].

In addition to vesiculoviruses, many other viruses in the *Rhabdoviridae* have been detected in phlebotomine sandflies, including Charleville, Inhangapi, Iriri, Niakha, Santa Barbara, and Sripur viruses (Table 10) [464,483,484,485,486]. However, none of these viruses have been isolated from phlebotomine sandflies on more than five occasions and none have been assessed in vector competence experiments. Phlebotomine sandflies also harbor viruses various from the family *Peribunyaviridae*, including Caimito, Chilibre, Guamá, Rio Preto da Eva, Pacui, Santarém, Tapara, and Uriurana viruses [460,464,487,488]. None of these viruses have been isolated from more than 2 pools of phlebotomine sandflies, except for Pacui virus, which was recovered from 100 of 658 pools of 61,437 *Lutzomyia flaviscutellata* in Brazil [487]. The ability of phlebotomine sandflies to experimentally transmit these peribunyaviruses has not been evaluated.

### 12.2. Other Vertebrate-Infecting Viruses

Phlebotomine sandflies harbor several non-arboviral viruses capable of replicating in vertebrate cells, including Hedi virus (*Phenuiviridae*), Saboya virus (*Flaviviridae*), Santa Barbara virus (*Rhabdoviridae*), and Wuxiang virus (*Phenuiviridae*) (Table 10). Hedi virus was isolated from *Phlebotomus chinensis* in China and replicates in BHK-21 cells [489]. Saboya virus was originally isolated from a gerbil (*Tatera kempi*) in Senegal and later detected in other rodent species and hematophagous arthropods, including phlebotomine sandflies, in Senegal and Guinea [54,468,490,491,492]. Santa Barbara virus has been isolated on two occasions, from a pool of *Psychodidae* sandflies (species not specified) and a mouse (species not specified) in Brazil [464]. Wuxiang virus was isolated from 10 pools of *Phlebotomus chinensis* in China and is capable of replicating in BHK-21 cells [493].

### 12.3. Viruses Not Known to Infect Vertebrates

Several viruses with apparent insect-specific host ranges belonging to the families *Flaviviridae*, *Phenuiviridae*, and *Rhabdoviridae* have been identified in phlebotomine sandflies [494,495,496,497]. Viruses from other taxonomic groups have also been detected in sandflies and they too appear to be insect-specific [498,499].

**Table 10:** Arboviruses and other vertebrate-infecting viruses identified in field-collected phlebotomine sandflies ^1^.

Virus	Species Name	Abbreviation	Family	Recognized Arbovirus	^2^ Experimental Evidence of Sandfly-Borne Virus Transmission	Citation
Adana virus	*Phlebovirus adanaense*	ADAV	*Phenuiviridae*	Yes	^3^ -	[500]
Aguacate virus	*Phlebovirus aguacateense*	AGUV	*Phenuiviridae*	Yes	-	[460,501]
Alcube virus	*Phlebovirus alcubeense*	ACBV	*Phenuiviridae*	Yes	-	[502]
Ambé virus	*Phlebovirus ambeense*	ABEV	*Phenuiviridae*	Yes	-	[488,503]
Arbia virus (a subtype of Salehabad virus)	*Phlebovirus salehabadense*	ARBV	*Phenuiviridae*	Yes	-	[54,451]
Ariquemes virus (a subtype of Candiru virus)	*Phlebovirus candiruense*	CDUV	*Phenuiviridae*	Yes	-	[504]
Arrábida virus (a subtype of Sandfly fever Naples virus)	*Phlebovirus napoliense*	ARRV	*Phenuiviridae*	Yes	-	[505]
Balkan virus (a subtype of Sandfly fever Naples virus)	*Phlebovirus napoliense*	BALKV	*Phenuiviridae*	Yes	-	[506,507]
Bregalaka virus (a subtype of Salehabad virus)	*Phlebovirus salehabadense*	BREV	*Phenuiviridae*	Yes	-	[508]
Buenaventura virus	*Phlebovirus buenaventuraense*	BUEV	*Phenuiviridae*	Yes	-	[54,461,509]
Cacao virus	*Phlebovirus cacaoense*	CACV	*Phenuiviridae*	Yes	-	[54,460]
Caimito virus	*Pacuvirus caimitoense*	CAIV	*Peribunyaviridae*	Yes	-	[54]
Campana virus	*Phlebovirus campanaense*	CMAV	*Phenuiviridae*	Yes	-	[460,461]
Capira virus (a subtype of Punta Toro virus)	*Phlebovirus toroense*	CAPIV	*Phenuiviridae*	Yes	-	[461]
Carajas virus	*Vesiculovirus carajas*	CJSV	*Rhabdoviridae*	Yes	-	[481]
Chagres virus	*Phlebovirus chagresense*	CHGV	*Phenuiviridae*	Yes	-	[54,460],
Chandipura virus	*Vesiculovirus chandipura*	CHPV	*Rhabdoviridae*	Yes	Yes (transmission to mice)	[467,468,470,473,474,510]
Changuinola virus	*Orbivirus changuinolaense*	CGLV	*Sedoreoviridae*	Yes	-	[511]
Charleville virus	*Sripuvirus charleville*	CHVV	*Rhabdoviridae*	Yes	-	[483]
Chilibre virus	*Pacuvirus chilibreense*	CHIV	*Peribunyaviridae*	Yes	-	[54]
Corfou virus	*Phlebovirus corfouense*	CFUV	*Phenuiviridae*	Yes	-	[54]
Dashli virus	*Phlebovirus dashliense*	DASV	*Phenuiviridae*	Yes	-	[512]
Durania virus	*Phlebovirus duraniaense*	DRNV	*Phenuiviridae*	Yes	-	[503]
Fermo virus (a subtype of Sandfly fever Naples virus)	*Phlebovirus napoliense*	FERV	*Phenuiviridae*	Yes	-	[451]
Frijoles virus	*Phlebovirus limboense*	FRIV	*Phenuiviridae*	Yes	-	[54,513]
Granada virus (a subtype of Sandfly fever Naples virus)	*Phlebovirus napoliense*	GRV; GRAV	*Phenuiviridae*	Yes	-	[514]
Guamá virus	*Orthobunyavirus guamaense*	GMAV	*Peribunyaviridae*	Yes	-	[54,464,487,515]
Hedi virus	*Phlebovirus hediense*	HEDV	*Phenuiviridae*	-	-	[489]
Inhangapi virus	*Arurhavirus inhangapi*	INHV	*Rhabdoviridae*	Yes	-	[484]
Iriri virus	*Curiovirus iriri*	IRIRV	*Rhabdoviridae*	Yes	-	[307]
Isfahan virus	*Vesiculovirus isfahan*	ISFV	*Rhabdoviridae*	Yes	-	[516,517]
Ixcanal virus	*Phlebovirus ixcanalense*	IXCV	*Phenuiviridae*	Yes	-	[503]
Joá virus (a subtype of Frijoles virus)	*Phlebovirus limboense*	JOAV	*Phenuiviridae*	Yes	-	[488]
Karimabad virus	*Phlebovirus karimabadense*	KARV	*Phenuiviridae*	Yes	-	[54,441,518]
Koutango virus	*Orthoflavivirus koutangoense*	KOUV	*Flaviviridae*	Yes	-	[519]
Maraba virus	*Vesiculovirus maraba*	MARV	*Rhabdoviridae*	Yes	-	[481]
Medjerda Valley virus	*Phlebovirus medjerdaense*	MVV	*Phenuiviridae*	Yes	-	[520]
Morreton virus	*Vesiculovirus morreton*	MORV	*Rhabdoviridae*	Yes	-	[307]
Munguba virus	*Phlebovirus mungubaense*	MUNV	*Phenuiviridae*	Yes	-	[488]
Nique virus	*Phlebovirus niqueense*	NIQV	*Phenuiviridae*	Yes	-	[504]
Oriximiná virus	*Phlebovirus oriximinaense*	ORXV	*Phenuiviridae*	Yes	-	[504]
Pacui virus	*Pacuvirus pacuiense*	PACV	*Peribunyaviridae*	Yes	-	[487]
Perinet virus	*Vesiculovirus perinet*	PERV	*Rhabdoviridae*	Yes	-	[54]
Punique virus	*Phlebovirus puniqueense*	PUNV	*Phenuiviridae*	Yes	-	[439,521,522,523]
Punta Toro virus	*Phlebovirus toroense*	PTV	*Phenuiviridae*	Yes	-	[54,461]
Radi virus	*Vesiculovirus radi*	RADV	*Rhabdoviridae*	Yes	-	[54]
Rift Valley fever virus	*Phlebovirus riftense*	RVFV	*Phenuiviridae*	Yes	Yes (transmission to hamsters)	[456] ^4^ Genbank Accession No. ON856673
Rio Preto da Eva virus	*Pacuvirus evaense*	RPEV	*Peribunyaviridae*	Yes	-	[524]
Saboya virus	*Orthoflavivirus saboyaense*	SABV	*Flaviviridae*	-	-	[54,468,490,492]
Saddaguia virus (a subtype of Sandfly fever Naples virus)	*Phlebovirus napoliense*	SADV	*Phenuiviridae*	Yes	-	[462]
Salehabad virus	*Phlebovirus salehabadense*	SALV	*Phenuiviridae*	Yes	-	[525]
Sandfly fever Naples virus	*Phlebovirus napoliense*	SFNV	*Phenuiviridae*	Yes	Possibly (transmission of a virus, likely SFNV or SFSV, to human volunteers)	[54,443,526,527,528,529]
Sandfly fever Sicilian virus	*Phlebovirus siciliaense*	SFSV	*Phenuiviridae*	Yes	Possibly (transmission of a virus, likely SFNV or SFSV, to human volunteers)	[54,422,431,440,441,442,443]
Sandfly fever Turkey virus	^5^ none	SFTV	*Phenuiviridae*	Yes	-	[417,428]
Santa Barbara virus	*Arurhavirus santabarbara*	SBAV	*Rhabdoviridae*	-	-	[464]
Santarém virus	none	STMV	*Peribunyaviridae*	Yes	-	[54]
Tapará virus	*Phlebovirus taparaense*	TPRV	*Phenuiviridae*	Yes	-	[488]
Tehran virus	*Phlebovirus tehranense*	THEV	*Phenuiviridae*	Yes	-	[54,441,530]
Toscana virus	*Phlebovirus toscanaense*	TOSV	*Phenuiviridae*	Yes	-	[425,428,450,531]
Turuna virus	*Phlebovirus turunaense*	TUAV	*Phenuiviridae*	Yes	-	[504]
Uriurana virus	*Phlebovirus uriuranaense*	URIV	*Phenuiviridae*	Yes	-	[488]
Vesicular stomatitis Alagoas virus	*Vesiculovirus alagoas*	VSAV	*Rhabdoviridae*	Yes	Yes (transmission to mice)	[482]
Vesicular stomatitis Indiana virus	*Vesiculovirus indiana*	VSIV	*Rhabdoviridae*	Yes	Yes (transmission to hamsters)	[54,80,477]
Vesicular stomatitis New Jersey virus	*Vesiculovirus newjersey*	VSNJV	*Rhabdoviridae*	Yes	Yes (transmission to hamsters)	[80,475,476,479]
Wuxiang virus	none	WUXV	*Phenuiviridae*	-	-	[493,532]
Yug Bogdanovac virus	*Vesiculovirus bogdanovac*	YBV	*Rhabdoviridae*	Yes	-	[54]
Zaba virus (a subtype of Salehabad virus)	*Phlebovirus salehabadense*	ZABAV	*Phenuiviridae*	Yes	-	[508]
Zerdali virus	*Phlebovirus zerdaliense*	ZERV	*Phenuiviridae*	Yes	-	[533]

^1^ Not an exhaustive list. ^2^ No. ^3^ Excludes mechanical transmission. ^4^ This GenBank entry was obtained by searching the “Nucleotide” section of the NCBI database using the terms “Rift Valley fever virus” and “Phlebotomus”. ^5^ Not recognized by the International Committee on Taxonomy of Viruses.

## 13. Tabanids

Tabanids (*Tabanidae*) are hematophagous flies, belonging to 4665 species, 177 genera, and five subfamilies (*Adersinnae, Chrysopsinae*, *Pangoniinae*, *Scepsidinae*, and *Tabaninae*) [534]. Tabanids have a wide geographic distribution and are found in almost all habitats. The best known and most widely studied tabanids are horse flies (*Tabanus* spp.) and deer flies (*Chrysops* spp.), although sometimes all tabanids are referred to as horse flies [535]. Horse flies and deer flies are nuisance biters of humans, livestock, and wildlife and are also mechanical vectors of several viruses of veterinary importance [536,537,538,539,540]. Moreover, one recognized arbovirus and other several vertebrate-infecting viruses have also been detected in tabanids (Table 11).

### 13.1. Recognized Arboviruses

Tabanids are not regarded as biological vectors of arboviruses, although Jamestown Canyon virus (JCV; *Peribunyaviridae*), a recognized arbovirus, was isolated from orange-sided horseflies (*Hybomitra lasiophthalma*) and ring-clawed deerflies (*Chrysops cincticornis*) in Wisconsin, the U.S. [541]. The ability of JCV to replicate in *C. cincticornis* and *H. lasiophthalma* has not been assessed, but JCV and Keystone virus (*Peribunyaviridae*) could not replicate in greenhead horse flies (*Tabanus nigrovittatus*) or deer flies (*Chrysops atlanticus*, *Chrysops fuliginosus*, and *Chrysops obsoletus*) under laboratory conditions [542].

**Table 11:** Arboviruses and other vertebrate-infecting viruses identified in field-collected tabanids.

Virus	Species Name	Abbreviation	Family	Recognized Arbovirus	^1^ Experimental Evidence of Tabanid-Borne Virus Transmission	Citation
Jamestown Canyon virus	*Orthobunyavirus jamestownense*	JCV	*Peribunyaviridae*	Yes	^2^ -	[541]
Kamenushka Hybomitra narna-like virus	^3^ none	KHNV	*Narnaviridae*	-	-	[543]
Medvezhye Chrysops ifla-like virus	none	MCIV	*Iflaviridae*	-	-	[543]
Medvezhye Chrysops narna-like virus 2	none	MCNV2	*Narnaviridae*	-	-	[543]
Medvezhye pound Haematopota permuto-like virus	none	MHPV	*Permutotetraviridae*	-	-	[543]
Medvezhye tabanus Toti-like virus	none	none assigned	*Totiviridae*	-	-	[543]
Melisia Chrysops solemo-like virus	none	MCSV	*Solemoviridae*	-	-	[543]
Volxa Hybomitra toti-like virus	none	VHTV	*Totiviridae*	-	-	[543]
Xanka Hybomitra negev-like virus	none	XHNV	unclassified	-	-	[543]

^1^ Excludes mechanical transmission. ^2^ No. ^3^ Not recognized by the International Committee on Taxonomy of Viruses.

### 13.2. Other Vertebrate-Infecting Viruses

The viromes of *Chrysops*, *Haematopota Hybomitra*, and *Tabanus* spp. tabanids from Russia were characterized, resulting in the detection of many novel viruses and one recognized virus (Big Sioux River virus; *Dicistroviridae*) [543]. Big Sioux River virus and eight of the newly discovered viruses were able to replicate in pig embryo kidney (PEK) cells, but it is unknown whether they are biologically transmitted by tabanids to vertebrates (Table 11).

### 13.3. Viruses Not Known to Infect Vertebrates

Tabanus rufidens flavivirus (*Flaviviridae*) was first identified in horse flies (*Tabanus rufidens*) in Japan, but it is most closely related phylogenetically to insect-specific flaviviruses and cannot replicate in BHK-21 cells [544]. Other viruses with apparent insect-specific host ranges have also been detected in tabanids [358,543].

## 14. Triatomines

Triatomines (also known as kissing bugs) are obligate blood-feeders belonging to the subfamily *Triatominae* (*Reduviidae*) [545,546,547]. The subfamily contains 14 genera and at least 140 species. Triatomines primarily occur in the Americas but are also present in parts of Africa and Asia. Triatomines primarily feed on vertebrate blood but occasionally feed on the hemolymph of arthropods and are best known as the vectors of *Trypanosoma cruzi*, the protozoan that causes Chagas disease [548,549].

### 14.1. Recognized Arboviruses

Triatomines are not considered to be arboviral vectors, although SLEV and VEEV persisted for 30 and 124 days, respectively, in triatomines (*Rhodnius prolixus*) that had fed upon viremic mice [550]. Fifteen days post-engorgement, *R. prolixus* transmitted VEEV, but not SLEV, to mice. SLEV and VEEV have never been detected in field-collected triatomines.

### 14.2. Other Vertebrate-Infecting Viruses

No vertebrate-infecting viruses have been detected in field-collected triatomines.

### 14.3. Viruses Not Known to Infect Vertebrates

The first virus to be detected in naturally infected triatomines is Triatoma virus (*Dicistroviridae*), which appears to have a host range restricted to triatomines [551,552,553,554]. Other triatomine-associated viruses have since been discovered, including Meccus longipennis virus 1 (*Iflaviridae*), Drosophila melanogaster Nora virus (*Noraviridae*), and Rhodnius prolixus viruses 1 to 4 (unclassified), but none are known to replicate in vertebrates [555,556].

## 15. Tsetse Flies

The family *Glossinidae* comprises exclusively large, blood-feeding insects, known as tsetse flies. Approximately 31 species and subspecies are recognized, and all occur throughout much of sub-Saharan Africa [557,558,559]. These flies are vectors of trypanosome parasites, which cause sleeping sickness (or human African trypanosomiasis) in humans and nagana (or animal African trypanosomiasis) in livestock. Tsetse flies are not recognized arboviral vectors and are not known to harbor any vertebrate-infecting viruses. However, several viruses with apparent arthropod-specific host ranges have been detected in tsetse flies [560,561,562].

## 16. Conclusions

In conclusion, growing evidence suggests that lesser-studied hematophagous arthropods play important roles in arbovirus transmission. Notably, at least 132 recognized species/subtypes of arboviruses have been detected in non-mosquito, non-tick hematophagous arthropods (Table 2, Table 3, Table 4, Table 5, Table 6, Table 7, Table 8, Table 9, Table 10 and Table 11). Nonetheless, significant gaps remain in our understanding of their involvement in arboviral transmission. It is particularly important that we further investigate arthropods known or suspected to be epidemiologically important arbovirus vectors, namely, blackflies, cimicids, *Culicoides* midges, and phlebotomine sandflies. For example, blackflies are competent vectors of VSV under laboratory conditions and a high prevalence of VSV has been detected in blackflies during epizootics, but their role in the natural transmission of VSV is unclear. It is not known whether the majority of VSV-infected blackflies collected during outbreaks acquired the virus by biological or mechanical transmission. Further, it is not known whether blackflies contribute to the maintenance of VSV during non-epidemic periods or if they transmit the virus to wildlife. Additionally, a definitive reservoir host of VSV has yet to be identified. As such, blackflies have not been conclusively identified as epidemiologically important biological vectors of VSV and are often referred to as *suspected* or *incriminated* vectors. Experimental studies specifically designed to resolve these uncertainties are needed. Similarly, there is strong evidence that cimicids are the principal vectors of FMV; however, they cannot be classified as epidemiologically important vectors without field data demonstrating consistent FMV infection in wild cimicid populations. Addressing this knowledge gap will require targeted field investigations within the known geographic range of FMV.

*Culicoides* midges have been implicated as epidemiologically important biological vectors of several arboviruses, but additional research is required to fully characterize their role in transmission. Notably, there is a paucity of transmission experiments. For instance, *Culicoides* midges are considered primary vectors of AHSV, yet only three transmission experiments have been performed. Two studies used a natural host, but only one (an unpublished study) demonstrated successful transmission. Similarly, *Culicoides* midges are believed to be the primary vectors of AKAV, although most experimental infection studies focused on replication and dissemination rather than actual transmission. The exception is a study involving *Culicoides* species found outside the endemic range of AKAV. Another apparent *Culicoides*-borne arbovirus is BEFV, but successful experimental transmission of the virus is yet to be reported. EHDV is also likely transmitted primarily by *Culicoides* midges; however, most vector competence experiments have focused on North American *Culicoides* species and EHDV serotypes, leaving other species and serotypes unexplored. Transmission of MDV to uninfected blood suspensions by *Culicoides* midges has been documented, but transmission experiments involving vertebrate hosts have not been performed. *Culicoides* midges can experimentally transmit OROV to hamsters, but a high prevalence of OROV in field-collected *Culicoides* midges has not been consistently reported. Finally, SBV is assumed to be primarily *Culicoides*-borne, but all experimental infection studies have focused on replication and dissemination, without investigating transmission. These gaps highlight the need for well-designed transmission studies to clarify the role of *Culicoides* midges in the natural transmission cycles of these viruses.

Phlebotomine sandflies are suspected vectors of several phleboviruses, but their role in arbovirus transmission remains poorly characterized. Although numerous phleboviruses have been detected in phlebotomine sandflies, and some have been efficiently transmitted under laboratory conditions, no phlebovirus has been consistently detected in naturally infected sandflies and conclusively demonstrated to be transmitted to vertebrate hosts. As such, sandflies should be regarded as *suspected* or *incriminated* vectors of phleboviruses rather than epidemiologically important vectors. For example, phlebotomine sandflies are suspected to be the principal vectors SFNV, SFSV, and TOSV, all of which are significant human pathogens, yet comprehensive vector competence data are lacking. In a study performed during World War II, phlebotomine sandflies were shown to transmit a phlebovirus to soldier volunteers; however, the virus, while suspected to SFNV or SFSV, was never definitively identified. While experimental studies have shown that sandflies can support the replication and dissemination of TOSV, their ability to transmit the virus has not been tested. In addition to phleboviruses, sandflies are also suspected vectors of several vesiculoviruses, including CHPV. Although phlebotomine sandflies can transmit CHPV to mice under laboratory conditions, the insects were infected by intrathoracic inoculation, which, unlike oral challenge, bypasses the natural midgut barrier.

Two arthropod groups discussed in this review, namely, fleas and mites, may play a minor role in the biological transmission of arboviruses. Fleas have been implicated as minor vectors of TBEV based on two studies reporting the isolation of TBEV from multiple flea species and two other studies demonstrating experimental transmission by fleas. TBEV has also been shown to persist in fleas for up to six days. However, a key limitation of these studies is the lack of viral titer quantification, making it unclear whether TBEV actively replicates in fleas. In contrast, TBEV has been repeatedly isolated from field-collected ticks and efficiently transmitted by ticks under laboratory conditions, firmly establishing ticks as their primary vectors. Mites have been implicated as minor vectors of HTNV, with multiple studies reporting the isolation of the virus from field-collected mites and at least four studies demonstrating experimental transmission. Although HTNV is not officially classified as an arbovirus, its persistence in mites for up to 168 days indicates possible replication. Further studies are needed to determine whether HTNV titers increase over time in mites and whether the virus replicates in mite-derived cell lines. SLEV, EEEV, and WEEV have also been isolated from field-collected mites and transmitted by these arthropods under laboratory conditions, but transmission has either been inefficient or unable to be verified in follow-up studies, and it is firmly established that mosquitoes are their primary vectors. For all other arthropod groups (bat flies, hippoboscid flies, lice, muscid flies, tabanids, triatomines, and tsetse flies), there is insufficient evidence to suggest that they are biological vectors of arboviruses because none have been shown to transmit arboviruses under experimental conditions.

As previously mentioned, all known arboviruses, except for ASFV, have RNA genomes. RNA viruses evolve more rapidly than DNA viruses because their RNA-dependent RNA polymerases are inherently error-prone and lack proof-reading capabilities, resulting in higher mutation rates and, subsequently, an increased likelihood of adapting to two distinct biological environments (arthropod vectors and vertebrate hosts) [563,564,565,566,567]. Additionally, many RNA viruses have segmented genomes, allowing for reassortment, which further promotes genetic diversity and adaptation. In this regard, most arboviruses detected in *Culicoides* spp. midges, and phlebotomine sandflies belong to the *Peribunyaviridae* and *Sedoreoviridae*. The rapid evolutionary dynamics of RNA viruses likely contribute to the fact that the reservoirs hosts of many of the viruses discussed in this review remain unknown.

ZOVER is a database of zoonotic and vector-borne viruses launched in 2021 [568]. It integrates two previously established databases of bat- and rodent-associated viruses and compiles current information on viruses associated with mosquitoes and ticks. However, no equivalent resources exists for the other arthropod groups discussed in this review. This lack of a centralized database for arboviruses detected in non-mosquito, non-tick vectors presents a significant gap in our ability to identify underrecognized or emerging transmission cycles that may threaten human and animal health. Comprehensive risk assessments depend on a thorough understanding of all potential vector species. In the absence of data on these lesser-studied arthropods, our capacity to model transmission dynamics, forecast outbreaks, and implement effective vector control measures is reduced. Advancing our knowledge of the vector competence, host associations, and ecological roles of these groups is critical for a more complete understanding of arbovirus transmission and for strengthening both medical and veterinary preparedness.
